# Nuclear localization of TET2 requires β-catenin activation and correlates with favourable prognosis in colorectal cancer

**DOI:** 10.1038/s41419-023-06038-x

**Published:** 2023-08-24

**Authors:** Changpeng Li, Jingcai He, Fei Meng, Fuhui Wang, Hao Sun, Huizhong Zhang, Linna Dong, Mengdan Zhang, Qiaoran Xu, Lining Liang, Yuan Li, Tingting Yang, Meiai He, Tao Wang, Jiechun Lin, Jiaqi Sun, Qiuling Huang, Lin Guo, Xiaofei Zhang, Shijuan Mai, Hui Zheng

**Affiliations:** 1 Guangdong Provincial Key Laboratory of Stem Cell and Regenerative Medicine, GIBH-CUHK Joint Research Laboratory on Stem Cell and Regenerative Medicine, Guangzhou Institutes of Biomedicine and Health, Chinese Academy of Sciences, Guangzhou, 510530 China; 2 Centre for Regenerative Medicine and Health, Hong Kong Institute of Science & Innovation, Chinese Academy of Sciences, Hong Kong SAR, China; 3 Key Laboratory of Biological Targeting Diagnosis, Therapy and Rehabilitation of Guangdong Higher Education Institutes, The Fifth Affiliated Hospital of Guangzhou Medical University, Guangzhou, 510799 China; 4 University of Chinese Academy of Sciences, Beijing, 100049 China; 5 University of Science and Technology of China, Hefei, Anhui 230026 China; 6 Sun Yat-sen University Cancer Center, Guangzhou, 510060 China; 7 Joint School of Life Sciences, Guangzhou Medical University, Guangzhou, 511436 China; 8 Bioland Laboratory (Guangzhou Regenerative Medicine and Health Guangdong Laboratory), Guangzhou, 510700 China

**Keywords:** Tumour biomarkers, Prognostic markers

## Abstract

Mutation-induced malfunction of ten-eleven translocation methylcytosine dioxygenase 2 (TET2) is widely reported in haematological malignancies. However, the role of TET2 in solid cancers, including colorectal cancer (CRC), is unclear. Here, we found that TET2 malfunction in CRC is mostly due to decreased nuclear localization and that nuclear localization of TET2 is correlated with better survival of patients. To explore the underlying mechanisms, 14 immortalized solid tumour cell lines and 12 primary CRC cell lines were used. TET2 was mostly detected in the nucleus, and it induced significant DNA demethylation and suppressed cell growth by demethylating *RORA* and *SPARC* in cell lines like SW480. While in cell lines like SW620, TET2 was observed in the cytosol and did not affect DNA methylation or cell growth. Further examination with immunoprecipitation–mass spectrometry illustrated that β-catenin activation was indispensable for the nuclear localization and tumour suppression effects of TET2. In addition, the β-catenin pathway activator IM12 and the TET2 activator vitamin C were used simultaneously to enhance the effects of TET2 under low-expression conditions, and synergistic inhibitory effects on the growth of cancer were observed both in vitro and in vivo. Collectively, these data suggest that β-catenin-mediated nuclear localization of TET2 is an important therapeutic target for solid tumours.

## Introduction

In patients with haematopoietic diseases, including cancer, mutation of ten-eleven translocation methylcytosine dioxygenase 2 (*TET2*) is one of the most commonly observed genetic abnormalities [1]. TET2 belongs to the TET family of proteins that catalyse the conversion of 5-methylcytosine (5mC) to 5-hydroxymethylcytosine (5hmC). Various studies have demonstrated that mutation-induced dysfunction of TET2 leads to deregulation of haematopoietic cell lineages and subsequent development of myeloid abnormalities. The frequency of *TET2* mutations is approximately 20–35% for myelodysplastic syndrome [2], 12–34% for acute myeloid leukaemia [3], and 2–33% for lymphoid malignancies [4]. TET2 mutations cooperate with *JAK2, ASXL1, SRSF2* and *KRAS* mutations to drive the development of various haematological malignancies [5–8]. In terms of solid tumours, several papers have reported decreased expression of TET2 in cancer tissues [9–11] and the nuclear loss of TET2 in colorectal cancer tissues [12]. However, the underlying mechanism of TET2’s role in solid cancer and the exact regulation of TET2’s cellular localization are largely unknown.

Unlike in haematological malignancies, solid tumour cells proliferate by attaching to a substratum and assuming either an epithelial or mesenchymal state [13]. Due to different cell states, solid tumours may present complex cell‒cell and cell–matrix interaction statuses [14]. These interactions of solid tumours are crucial factors that determine tumour development status [15], which accounts for the much more complex pathogenesis of solid tumours than haematological cancers. More importantly, epithelial–mesenchymal transition (EMT) and WNT pathway activation are frequently observed in advanced solid tumours when tumour growth reaches its limits [16]. These pathways are crucial for tumour growth, metastasis, recurrence and patient survival [17] .

β-Catenin is encoded by the *CTNNB1* gene, and malfunctioning of β-catenin signalling has been implicated in tumorigenesis, tumour progression, recurrence, and chemoresistance in solid tumours, especially colorectal cancer (CRC) [18]. Deactivated β-catenin is an important adherens junction protein maintaining cell-to-cell adhesion. Once activated, the cell adhesion complex is released, and the epithelial cells transform into a more mesenchymal phenotype. In the cytoplasm, β-catenin forms a complex with T-cell factor/lymphoid enhancer factor family 1 (TCF/LEF-1) and then translocates to the nucleus and induces WNT-β-catenin pathway activation. All these findings strongly support that activated β-catenin is the central protein of both EMT and the WNT/β-catenin pathway and is closely associated with tumour status maintenance.

In this study, we explored the dysfunction of TET2 in colorectal cancer and observed that the subcellular localization and DNA demethylation activity of TET2 are critical for CRC progression. Moreover, the subcellular localization and DNA demethylation activity of TET2 are connected with EMT and WNT-β-catenin pathway activation. Our studies may provide a much-needed rationale for the design of therapeutic interventions targeting solid tumours, especially CRC.

## Results

### TET2 plays critical roles during the progression of CRC

**Fig. 1:**
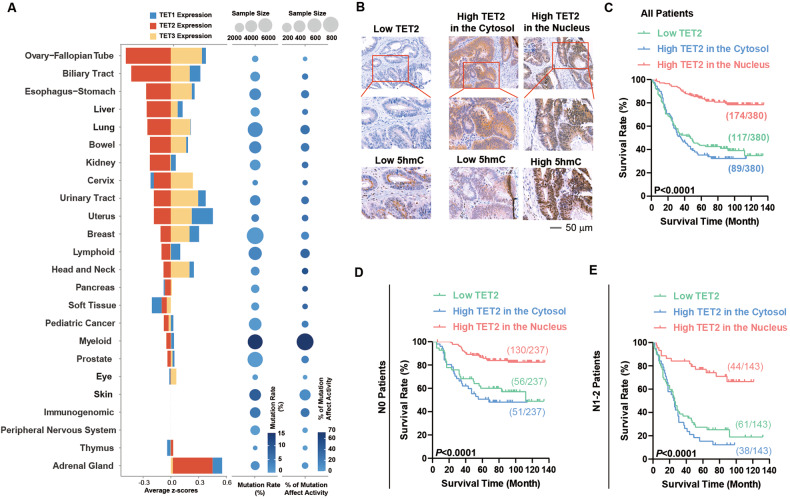
The mechanism of TET2 dysfunction in solid tumours is different from that in blood cancers. **A** TET2 mutation analysis and TET1/2/3 expression analysis in different cancer types from the TCGA database. **B**–**E** Representative IHC images of TET2 and 5hmC in CRC samples (**B**). Kaplan–Meier survival plots for the three indicated categories of all 380 patients (**C**), the 237 patients who were classified into N0 (**D**), and the 143 patients who were classified into N1-2 (**E**). All experiments were repeated at least 5 times (*n* ≥ 5). Additional statistical information is provided in Supplementary Table S8.

To identify the overall landscape of *TET2* malfunction, we examined the mutation and expression of *TET2* in cancers originating from different organs in the TCGA database (Fig. 1A). As previously reported, a high frequency of gene mutation was identified in lymphoid and myeloid tumours, and approximately 67% of these mutations were located in the catalytic domain, affecting the DNA demethylation activity of TET2 [19]. For solid tumours, the frequency of gene mutations (1.93%, 815cases of solid tumor were mutated in a total of 42224 cases) and the percentage of mutations that affected TET2 activity (18.7%, 152 cases of mutation located in the catalytic domain in a total of 815 cases) were much lower. In contrast, low levels of *TET2* mRNA were identified in several solid tumours, including CRC (Fig. 1A). Therefore, the mechanism of *TET2* dysfunction in solid tumours may be different from that in blood cancers.

We then evaluated the protein level of TET2 via immunohistochemistry (IHC) in two different cohorts, a CRC tissue array (Shanghai-Taizhou cohort, 194 patients) and CRC paraffin sections (Guangzhou cohort, 186 patients). As shown by IHC, 117 samples had low TET2 expression out of a total of 380 samples tested. Kaplan–Meier analyses revealed that patients with high levels of TET2 showed significantly better overall survival (OS) (*p* < 0.001) (Supplementary Figure S1A-D & Table S1). In the 263 samples with high TET2 expression, nuclear localization of TET2 was observed in 174 samples. In the other 89 samples, TET2 protein was only observed in the cytosol (Table S1). Then, 5-hydroxymethylcytosine (5hmC) was stained in serial sections of CRC tissues from the same areas. We found that patients with high levels of TET2 protein located in the nucleus exhibited higher levels of 5hmC, and patients with low levels of TET2 protein or high levels of TET2 protein located in the cytoplasm exhibited low levels of 5hmC (Fig. 1B & Supplementary Figure S1E). These results demonstrate that DNA demethylation activity is higher in patients with high levels of TET2 located in the nucleus than TET2 low expression and TET2 high level in cytoplasm.

Additional Kaplan–Meier analyses revealed that patients with high nuclear expression of TET2 showed better survival than those with low or cytosolic TET2 expression (*p* < 0.001), while patients with low TET2 expression and TET2 located in the cytosol had almost the same survival rate (Fig. 1C & Supplementary Figure S1F-G). Subsequent correlation analyses demonstrated that a high level of TET2 in the nucleus was negatively associated with lymph node invasion (*P* < 0.001), distant metastasis (*P* < 0.001) and Dukes’ stages (*P* < 0.001) (Supplementary Figure S1H & Table S2). In 72 samples with low TET2 expression with discernible subcellular location, TET2 located in cytosol also exhibited worse survival (Supplementary Figure S1I). Overall, these results demonstrate that *TET2* has tumour suppressive activities.

To further undermine the roles of TET2 nuclear localization in different stages of tumour progression, these samples were divided into two groups based on TNM staging: as indicated in Fig. 1D, E, patients without lymph node metastasis were classified into N0, and patients with node metastasis were classified into N1-2. The subcellular localization of TET2 was more important for the survival of N1-2 patients than for N0 patients, which suggested a stage-dependent effect of TET2 (Fig. 1D, E). Similar phenomena were not observed for different T and M stages. As demonstrated by multi-variant COX regression analysis in Tables S2, TET2 could still affect the prognosis of patients, even when the effects of tumor stage, lymph node metastasis and distant metastasis were excluded (Supplementary Table S2).

### Nuclear TET2 suppresses cancer cell growth and migration via DNA demethylation

To further study the role of TET2-mediated DNA demethylation activity in CRC, the catalytic domain of TET2 (Tet2CD) was exogenously expressed in 6 immortalized CRC cell lines, 12 primary cell lines established from freshly resected CRC and 8 immortalized cell lines established from other solid cancers (Supplementary Table S3). The growth-inhibitory effects of Tet2CD overexpression were then assessed. Sixteen cell lines with significant growth inhibition were classified into the “suppression” type. Ten cell lines with no growth inhibition were classified into the “no suppression” type (Supplementary Table S3).

**Fig. 2:**
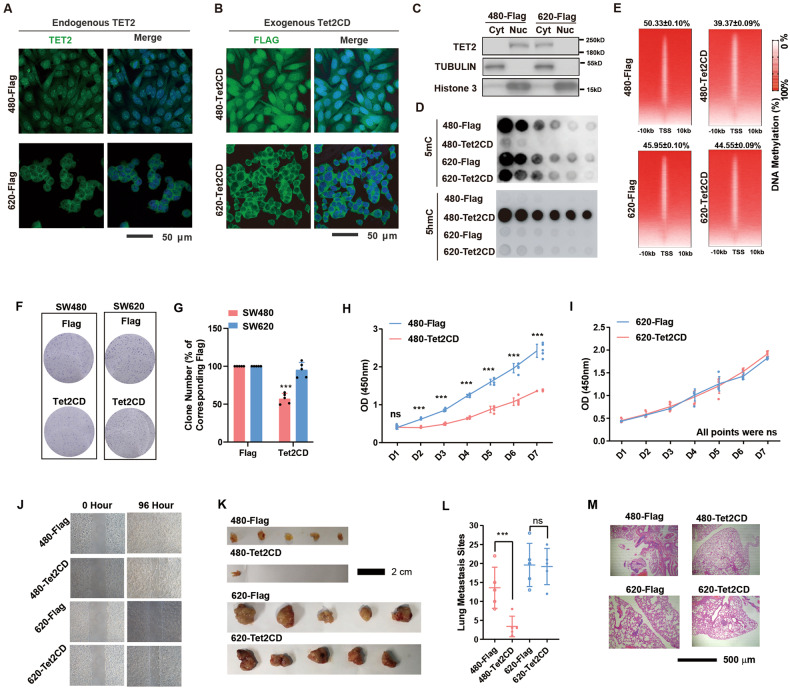
Nuclear localization is crucial for the DNA demethylation and tumour suppression activity of TET2 in solid cancer cell lines. **A**, **B** Immunofluorescence staining for both endogenous TET2 (**A**) and exogenous Tet2CD (**B**) in SW480 and SW620 cells. **C** Cytoplasm and nuclear protein fractionation analysis of indicated cells. **D**–**E** The DNA methylation levels in SW480 and SW620 cells expressing Flag or Tet2CD were determined by dot blotting (**D**) and WGBS (**E**). **F**–**J** The performance of SW480 and SW620 cells expressing Flag or Tet2CD in colony formation (**F**–**G**), cell growth (**H**–**I**) and wound healing (**J**) assays was determined. Note that the effects of Tet2CD were only observed in SW480 cells. **K**–**M** The indicated cell lines were transplanted into nude mice. The sizes of subcutaneous tumours (**K**) and lung metastasis (**L**, **M**) were measured at week 4. All experiments were repeated at least 5 times (*n* ≥ 5), except for sequencing experiments. Additional statistical information is provided in Supplementary Table S8.

Of all cell lines tested, SW480, of the “suppression” type, and SW620, of the “no suppression” type, were unique in the sense that both were isolated from the same CRC patient [20] but responded differentially to Tet2CD overexpression. In SW480 cells, both endogenous and exogenous TET2 localized to the nucleus. In contrast, TET2 was cytosolic in SW620 cells (Fig. 2A–C). Consistent with the different subcellular localizations of TET2, dot blotting and whole-genome bisulfite sequencing (WGBS) analysis revealed greater global DNA demethylation induced by Tet2CD in SW480 cells than in SW620 cells (Fig. 2D, E).

In addition, we found that Tet2CD impaired the growth and migration of SW480 cells but not SW620 cells both in vitro and in vivo (Fig. 2F–M). However, the other two members of the TET family, TET1 and TET3, did not exert this kind of effect (Supplementary Figure S2A-E), suggesting that this phenomenon is specific to TET2. More importantly, overexpression of full-length TET2 (Tet2FL) inhibited the growth of SW480 cells but not SW620 cells (Supplementary Figure S2F-K). Correspondingly, CRISPR‒Cas9-mediated *TET2* knockout stimulated the growth and migration of SW480 cells but not SW620 cells (Supplementary Figure S2L-Q). These results further confirm the hypothesis that TET2 inhibits the growth and migration of CRC cells by inducing DNA demethylation.

### *RORA* and *SPARC* are crucial downstream targets of TET2

**Fig. 3:**
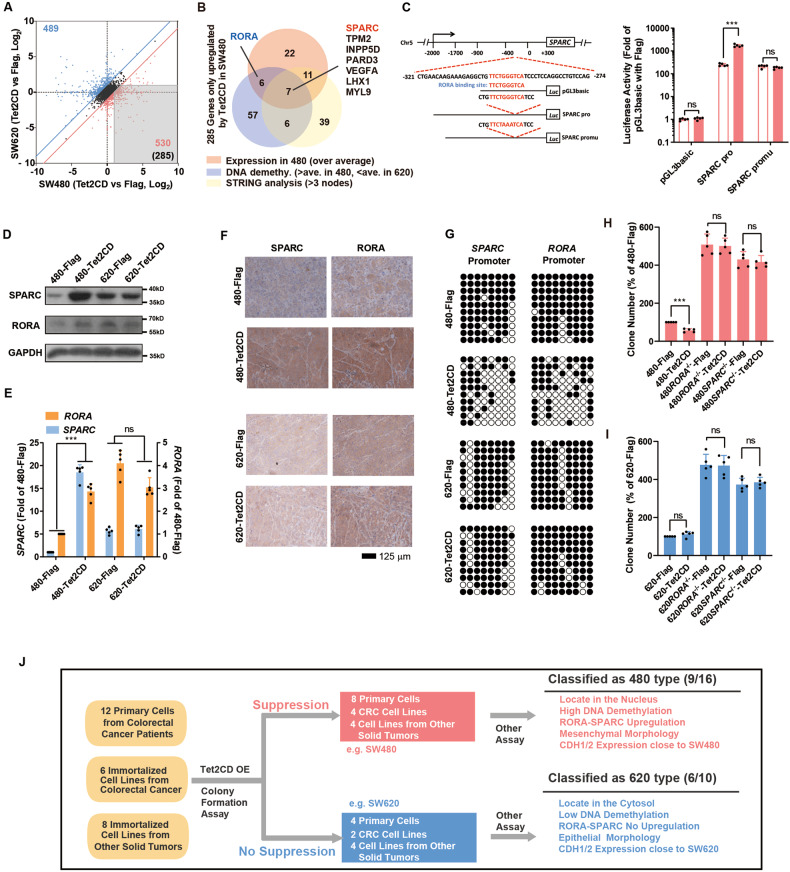
Nuclear localization of TET2 inhibits CRC progression by inducing DNA demethylation of *RORA* and *SPARC*. **A**, **B** The expression changes induced by Tet2CD overexpression in SW480 and SW620 cells are plotted together (**A**). As indicated in the grey area, 285 genes were upregulated by Tet2CD in SW480 cells only. The Venn diagram shows genes selected based on their expression levels, DNA demethylation levels, and interactions with other proteins (**B**). **C** *RORA* bound and activated the promoter of *SPARC* in HEK293T cells. **D**–**E** The protein (**D**) and mRNA levels (**E**) of RORA and SPARC were determined in SW480 and SW620 cells expressing Flag or Tet2CD. **F** The levels of RORA and SPARC were determined in SW480 and SW620 cells expressing Flag or Tet2CD four weeks after transplantation into nude mice. Higher levels of RORA and SPARC were observed in 480-Tet2CD than in 480-Flag. Such observations were not obtained in SW620 cells. **G** Demethylation on the promoters of *RORA* and *SPARC* was determined with bisulfite sequencing. Tet2CD decreased the methylation on the promoters of *RORA* and *SPARC* only in SW480 cells. **H**–**I** Colony-forming assay in SW480 and SW620 cells overexpressing TET2CD and/or RORA^-/-^ and SPARC^-/-^ cells; notably, Tet2CD failed to suppress the growth of RORA^-/-^ and SPARC^-/-^ SW480 cells. **J** Generalization of different categories of cell lines based on the functions of Tet2CD. Cell mesenchymal morphology was defined as low cell-cell interaction and high aspect ratio (over 1.5), while epithelial morphology was considered as high cell-cell interaction and low aspect ratio (below 1.5). All experiments were repeated at least 5 times (*n* ≥ 5), except for sequencing experiments. Additional statistical information is provided in Supplementary Table S8.

To evaluate the downstream targets of TET2, RNA-seq analysis of SW480-vector, SW480-TET2CD, SW620-vector and SW620-TET2CD cells was carried out, and we identified 285 genes that were upregulated by TET2CD in SW480 but not SW620 cells (Fig. 3A). Three additional criteria were also used to select candidate target genes for TET2: the expression level (elevated expression in SW480-Tet2CD), DNA demethylation status (significant DNA demethylation level change in SW480-Tet2CD but not in SW620-Tet2CD), and interactions with other proteins (STRING nodes>3) [21]. Finally, we selected 7 genes from the abovementioned 285 genes (Fig. 3B & Supplementary Table S4).

Among these genes, secreted protein acidic and cysteine rich (*SPARC*) and *RORA* stood out because of their suggested roles in CRC [22–24]. More importantly, after checking the promoter of *SPARC*, we found a RORA-binding motif in the promoter of SPARC. A promoter luciferase assay demonstrated that RORA is indeed an upstream regulator of *SPARC* (Fig. 3C). Given the unique relationship of RORA and SPARC, these molecules were analysed simultaneously in our following study.

Tet2CD overexpression significantly elevated the expression of RORA and SPARC at both the protein and mRNA levels in vivo and in vitro in SW480 cells but not in SW620 cells (Fig. 3D-F). Subsequent bisulfite sequencing revealed that Tet2CD overexpression induced significant DNA demethylation of CpG islands of the *RORA/SPARC* promoter in SW480 cells but not in SW620 cells (Fig. 3G). Similar phenomenon was observed in tissues with high TET2 activity (Figure S3A). Then, *RORA/SPARC* was knocked out in SW480 and SW620 cells, and Tet2CD failed to suppress the growth or migration of *RORA*^-/-^ and *SPARC*^-/-^ cells (Fig. 3H-I & Supplementary Figure S3B-D). In summary, *RORA* and *SPARC* are crucial downstream targets of TET2 that connect TET2 and its tumour suppression effects.

To further examine the function of TET2-mediated DNA demethylation of *SPARC* and *RORA* in other solid tumour cell lines, we examined the TET2 location, DNA demethylation activity and *RORA/SPARC* expression in these cells. Nine out of 16 “suppression”-type cell lines overexpressed Tet2CD located in the nucleus, induced high levels of DNA demethylation, and upregulated *RORA-SPARC*, similar to the observations in SW480 cells. Six out of 10 “no suppression”-type cell lines overexpressed TET2 located in the cytosol, induced low levels of DNA demethylation, and did not upregulate *RORA-SPARC*, similar to the observations in SW620 cells (Fig. 3J & Supplementary Table S3). Taken together, these findings indicate that TET2 inhibits tumour cell growth and metastasis by activating demethylation activity via regulation of *RORA-SPARC* expression.

### β-Catenin regulates TET2 activity by modulating TET2 nuclear localization

**Fig. 4:**
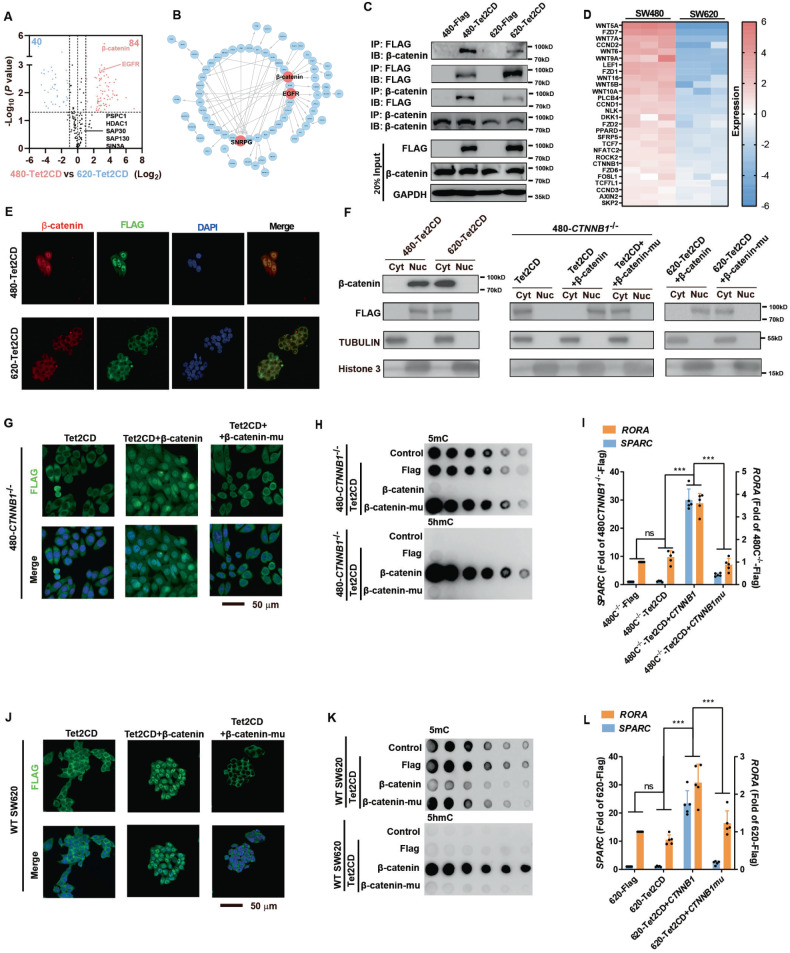
β-Catenin directly interacts with TET2 and regulates TET2 nuclear localization and DNA demethylation activity. **A** The proteins that interact with TET2 in SW480 and SW620 cells were identified via proteomic experiments. **B** Network of proteins that interact with TET2 at a higher intensity in SW480 cells. **C** The interaction between β-catenin and Tet2CD was confirmed by IP. **D** The expression of key factors in the WNT pathway in SW480 and SW620 cells was summarized after RNA-seq analysis. **E** IF analysis of the co- localization of Tet2CD and β-catenin in indicated cells. **F** Cytoplasm and nuclear protein fractionation assay was carried out in indicated cells and analysed with WB. **G**–**I** *CTNNB1*^-/-^ SW480 cells exhibited nuclear–cytosolic shuttling of overexpressed Tet2CD (**G**). *CTNNB1* knockout inhibited DNA demethylation (**H**) and reduced the expression of *RORA/SPARC* (**I**), effects that were reversed by wild-type β-catenin but not mutant β-catenin. **J**–**L** Overexpression of β-catenin, but not β-catenin-mu, in SW620 cells caused nuclear translocation of Tet2CD (**J**), induced global DNA demethylation (**K**) and increased the expression of RORA/SPARC (**L**). All experiments were repeated at least 5 times (*n* ≥ 5), except the sequencing experiments. Additional statistical information is provided in Supplementary Table S8.

To identify the regulators responsible for the different localizations and activities of TET2 in SW620 and SW480 cells, we performed proteomic experiments to detect any protein that may bind Tet2CD. A total of 148 proteins were found to significantly bind Tet2CD in SW480 cells, and 127 proteins were found to significantly bind Tet2CD in SW620 cells (Supplementary Figure S4A-B and Table S5). Several previously reported proteins, such as PSPC1, SIN3A, SAP30, and HDAC1 [25, 26], were identified and found to interact with TET2 at similar intensities in these two cell lines (Fig. 4A). More importantly, 84 proteins that interacted with TET2 at a higher intensity in SW480 cells were identified as the potential proteins responsible for the different activity of TET2 between SW480 and SW620 cells (Fig. 4A). Among these proteins, we noticed that β-catenin (encoded by the *CTNNB1* gene) was an important hub in the network of these 84 proteins, and β-catenin was selected for the following study (Fig. 4B).

We then validated that the interaction between TET2 and β-catenin indeed exists via immunoprecipitation studies (Fig. 4C & Figure S4C). Importantly, GO analysis of genes with higher expression in SW480 cells than in SW620 cells revealed that these genes were significantly related to the canonical WNT-β-catenin pathway (Supplementary Figure S4D-E). The following expression analysis indicated that these genes related to the WNT-β-catenin pathway exhibited higher expression in SW480 cells than in SW620 cells (Fig. 4D). In addition, IF analysis demonstrated the interaction of TET2 and β-catenin is higher in SW480 (Fig. 4E). All of these results suggest the potential contribution of β-catenin to the differential localization and activity of TET2 in SW480 and SW620 cells.

To further illustrate the importance of β-catenin, cell fractionation was carried out in SW480-Tet2CD and SW620-Tet2CD cells. β-catenin and Tet2CD were mainly located in cell nuclear in SW480-Tet2CD cells. In addition, *CTNNB1* was knocked out in SW480 cells (Supplementary Figure S4F). Knockout of *CTNNB1* resulted in shuttling of overexpressed Tet2CD from the nucleus to the cytosol, inhibited the DNA demethylation activity of Tet2CD and reduced the expression of *RORA/SPARC*, effects that could be reversed by wild-type β-catenin but not mutated β-catenin (without a nuclear translocation domain) [27] (Fig. 4F–I). Furthermore, overexpression of β-catenin, but not β-catenin-mu, in SW620 cells caused the translocation of Tet2CD from the cytosol to the nucleus, elevated global DNA demethylation and increased the expression of *RORA/SPARC* (Fig. 4F & Fig. 4J–L). The colony formation assay provided consistent results (Supplementary Figure S4G-H).

**Fig. 5:**
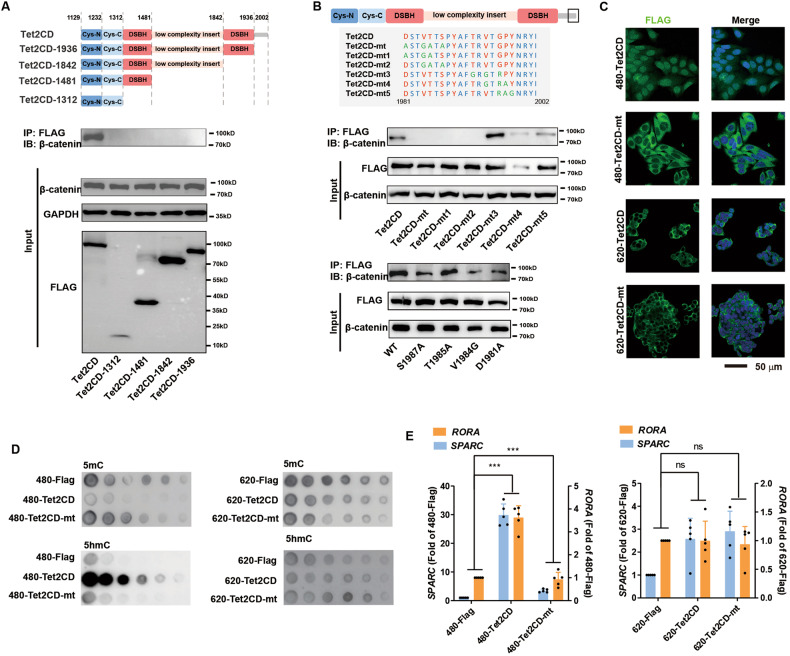
Key sites of TET2 were identified to be crucial for the interaction with β-catenin. **A** Various TET2 deletion constructs were expressed in 293 T cells. IP assays were used to determine whether they interacted with β-catenin. **B** Various TET2 mutants were expressed in 293T cells. Tet2CD-mt failed to interact with β-catenin. **C**–**D** Tet2CD or Tet2CD-mt was overexpressed in SW480 and SW620 cells. The localization of the overexpressed proteins (**C**) and the abilities of the proteins to induce global DNA demethylation (**D**) were determined. **E** Overexpression of Tet2CD-mt did not affect the expression of the *RORA-SPARC* axis. All experiments were repeated at least 5 times (*n* ≥ 5), except the sequencing experiments. Additional statistical information is provided in Supplementary Table S8.

To identify key sites of TET2 for its interaction with β-catenin, TET2 truncation constructs were created and used in immunoprecipitation assays. We found that the last 66 amino acids were critical for the TET2-β-catenin interaction (Fig. 5A). Additional mutation studies suggested that four conserved sites (D1981, V1984, T1985, and S1987) were important (Fig. 5B). When overexpressed in SW480 and SW620 cells, Tet2CD-mt (D1981A, V1984G, T1985A, and S1987A) failed to interact with β-catenin. Interestingly, nuclear protein lysates of Tet2CD-mt cells exhibited no DNA demethylation activity, but whole-cell protein lysates exhibited normal DNA demethylation activity, which indicated that Tet2CD-mt still had DNA demethylation activity (Supplementary Figure S4I-J). However, Tet2CD-mt localized to the cytosol and did not induce global DNA demethylation, nor did it increase the expression of *RORA-SPARC* (Fig. 5C–E & Supplementary Figure S4K). Consistently, Tet2CD-mt did not affect the growth of CRC cells (Supplementary Figure S4L). Therefore, β-catenin regulates the DNA demethylation activity of TET2 by modulating its nuclear localization via direct binding.

E-cadherin and β-catenin are well-known EMT markers that form a complex in epithelial cells. Although both SW480 and SW620 cells have epithelial characteristics, SW480 cells are more mesenchymal than SW620 cells based on a previously reported scoring system for EMT/MET [28] (Supplementary Figure S5A). To test whether EMT regulates the localization and activity of TET2 in SW620 and SW480 cells, we treated the cells with the widely used EMT activator and inhibitor TGFβ and RepSox, respectively [29]. TGFβ successfully induced EMT in SW620 cells, and RepSox induced MET in SW480 cells, which was confirmed by RNA-seq and cell migration via live cell tracing (Supplementary Figure S5A-C & Table S6). Subsequent assays demonstrated that RepSox induced TET2 shuttling from the nucleus to the cytosol in SW480 cells, which was accompanied by reduced TET2-dependent global DNA demethylation, impaired activation of the *RORA-SPARC* axis and accelerated growth of cancer cells; TGFβ exerted the opposite effects in SW620 cells (Supplementary Figure S5D-I & Table S6).

### Combination treatment with IM12 and vitamin C suppresses CRC progression

**Fig. 6:**
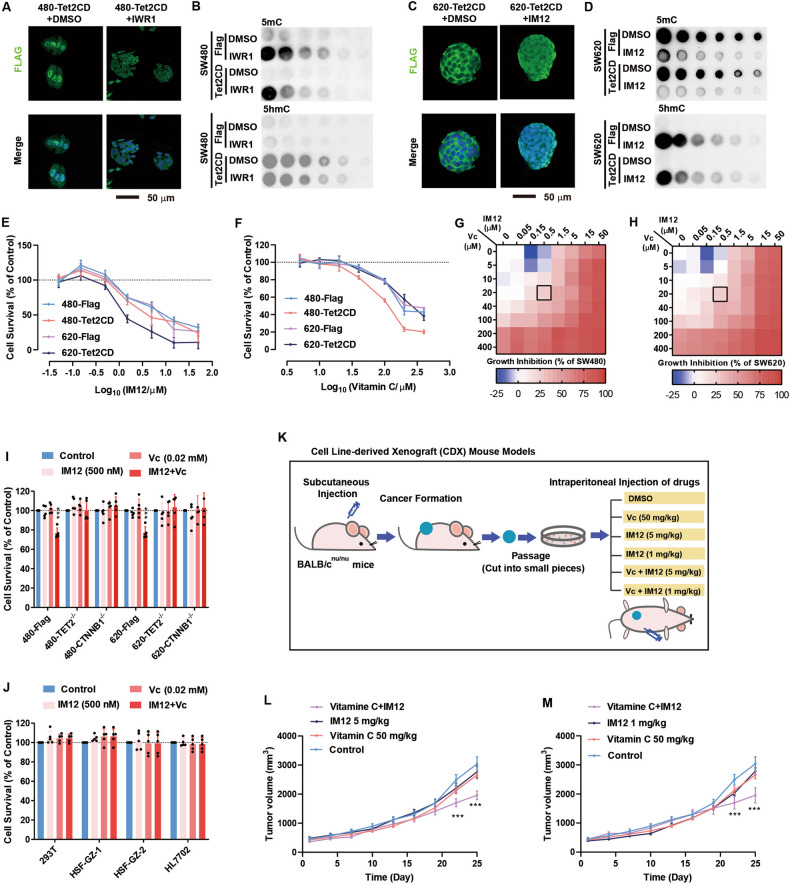
Vc and the β-catenin activator IM12 synergically inhibit tumour growth. **A**–**D** Compounds (β-catenin inhibitor IWR1, 25 μM; β-catenin activator IM12, 3.8 μΜ) were used to treat SW480 and SW620 cells overexpressing Tet2CD. The localization of Tet2CD was determined in SW480 (**A**) and SW620 (**C**) cells. A dot blotting analysis of DNA methylation in SW480 (**B**) and SW620 (**D**) cells was performed. **E**, **F** The concentration-dependent effects of IM12 (**E**) and Vc (**F**) were determined in SW480 and SW620 cells overexpressing Flag or Tet2CD. **G**, **H** IM12 and Vc (concentration indicated) were used to simultaneously or individually treat SW480 (**G**) and SW620 (**H**) cells. Cell growth was monitored. **I** IM12 (0.5 μM) and Vc (20 μM) were used simultaneously or individually to treat 480-Flag, 480-TET2^-/-^, 480-CTNNB1^-/-^, 620-Flag, 620-TET2^-/-^ and SW620-CTNNB1^-/-^ cells. Cell growth was monitored. **J** IM12 and Vc (concentration indicated) were used simultaneously or individually to treat several types of somatic cells. Cell growth was monitored. HSF and HL7702 represent human skin fibroblasts and hepatocytes, respectively. **K**–**M** CDX models prepared with SW620 cells were used to examine the effect of vitamin C (50 mg/kg), IM12 (1 or 5 mg/kg), or Vc plus IM12 on tumour growth (**K**). Vc and IM12 synergistically suppressed tumour cell growth (**L**, **M**). Additional statistical information is provided in Supplementary Table S8.

To further demonstrate the roles of β-catenin in regulating TET2 activity, the β-catenin inhibitor IWR1 and activator IM12 were used to treat SW480 and SW620 cells separately [30, 31]. In SW480 cells, IWR1 treatment resulted in the translocation of Tet2CD from the nucleus to the cytosol, impaired TET2-induced DNA demethylation, facilitated the growth of cancer cells and decreased the expression of *RORA* and *SPARC* (Fig. 6A, B and Supplementary Figure S6A-C). Correspondingly, in SW620 cells, IM12 led to cytosolic-nuclear shuttling, activation of global DNA demethylation, suppression of CRC cancer cell growth and enhanced expression of RORA and SPARC (Fig. 6C, D and Supplementary Figure S6D-E).

The notion that vitamin C (Vc) can be used to kill cancer cells has long been controversial during the past century. Vc has been widely reported as a strong activator of TET2 enzymatic activity [32, 33]. However, the clinical use of Vc requires a high dose, which raises safety concerns. Since IM12 is a novel activator of TET2 activity, we wondered whether IM12 may function as an anticancer agent. Indeed, IM12 and Vc inhibited cell growth in a concentration-dependent manner (Fig. 6E, F). Moreover, a synergistic effect was observed when we combined these two agents. Due to the synergistic effect, low levels of IM12 (0.5 μM) and Vc (20 mM) significantly suppressed cell growth to below 80% of the control level (Fig. 6G, H). These results demonstrate that IM12 may decrease the dose of Vc slightly required to kill tumour cells. This synergistic effect is significant but modest. However, such a synergistic effect was not observed in *TET2*^-/-^ or *CTNNB1*^-/-^ cells, which confirmed its dependency on the β-catenin-TET2 interaction (Fig. 6I). Similar effects were not observed in several mouse or human somatic cell lines (Fig. 6J), suggesting the potential use of IM12 and Vc together to treat cancer while future optimization is still needed to boost the synergistic effect.

We then prepared SW620 cell line-derived xenograft (CDX) models to examine the effect of Vc and IM12 in vivo (Fig. 6K). At the last two tested time points (Days 22 and 25), the tumour sizes in mice treated with both Vc and IM12 were smaller than those in the vehicle control and monotherapy groups (p < 0.001) (Fig. 6L, M). Therefore, Vc and IM12 synergistically suppress CRC progression both in vitro and in vivo. The synergistic effects are significant but modest.

In addition, the cell lines tested in Fig. 3J were used to test the synergistic effects of vitamin C and IM12 in suppressing cell growth. Similar synergic effects were also observed in 20 cell lines out of 26 total cell lines tested (Supplementary Figure S6F-G& Table S3). Therefore, the combination of Vc and IM12 may be used to treat other solid tumours and reduce the dosage of Vc.

## Discussion

The major form of TET2 malfunction in haematological cancers arises from gene mutation. However, our study shows that in solid tumours, the frequency of *TET2* mutation is low, and the malfunction of TET2 in solid tumours such as CRC can be achieved by loss of the nuclear localization of TET2.

In our study, we found that the difference in TET2 dysfunction between solid tumours and blood cancers is mainly due to differences in the cellular environment. In solid tumours, cells usually adhere to each other via adhesion complexes. E-cadherin and β-catenin are well-known members of the cell adhesion complex. However, B cells and T cells express genes related to cell‒cell and cell–substrate interactions, such as E-cadherin and N-cadherin, at relatively low levels [34]. Haematopoietic cancer cells grow mostly in suspension and cannot use E-cadherin to lock β-catenin in the membrane complex. In solid tumours, however, when cells sense EMT stimuli such as hypoxia, E-cadherin is degraded, which not only induces a mesenchymal phenotype but also releases β-catenin from the membranous complex. Therefore, the release of β-catenin from the cell adhesion complex is a basic cellular process involved in the development and progression of solid tumours [35]. However, no such phenomenon occurs in blood cancer cells.

In addition, released β-catenin from the adhesion complex may subsequently translocate from the cell membrane to the cytoplasm. For canonical Wnt signalling, β-catenin is the crucial nuclear effector. In the absence of Wnt ligands, cytoplasmic β-catenin is regulated by the destruction complex for final degradation. When the Wnt/β-catenin pathway is activated, cytoplasmic β-catenin translocates to the nucleus to activate the downstream transcriptional cascades of the Wnt pathway [36]. In solid tumours, especially in CRC, β-catenin activation plays crucial roles. However, the canonical β-catenin pathway is nearly undetectable in blood cancers [37].

As β-catenin is a crucial protein in both EMT and Wnt signalling and has diverse roles in many biological and pathological processes [38], the relationship between β-catenin and cancer progression seems very complex and even controversial. In our study, we found that elevated β-catenin translocates TET2 from the cytosol to the nucleus and suppresses tumour growth. Therefore, the activation of TET2 by EMT/WNT/β-catenin pathways is a kind of “side effect” or brake that impairs these key events in a feedback manner during tumour progression. Our study thus sheds light on the causes and mechanisms of TET2 dysfunction in solid tumours, which are quite different from those in haematological cancers. As demonstrated in the current study, nuclear β-catenin induces nuclear translocation of TET2 and enables TET2 to induce DNA demethylation, which subsequently suppresses cancer progression.

Previous studies have extensively reported that Vc activates TET2 activity [39]. Vc has also been used in the treatment of cancer for decades [40]. However, effective tumour prohibition requires high Vc doses, which are difficult to achieve under most circumstances and cause numerous side effects. It is reasonable to assume that the requirement of high doses of Vc is due to the cytosolic localization of TET2, whereas IM12 induces the nuclear translocation of TET2 to enable Vc to function at low doses. Here, we explored the possibility of treating solid tumours with Vc and the WNT/β-catenin pathway activator IM12 at low doses. IM12 and Vc promoted the nuclear localization and catalytic activity of TET2 and synergistically suppressed tumour cell growth in vivo and in vitro. The synergistic effects are significant but modest and more comprehensive study is needed to optimize the synergistic effects. In addition, the combination of IM12 and Vc may also suppress the growth of tumours with low TET2 expression, since Vc has a high ability to activate TET proteins.

However, current model of β-catenin and TET2 failed to work in a portion of tested cells. In pluripotent cells like ESCs (data not shown) and normal cells, we failed to observe the translocation of TET2 with β-catenin modulation. All of these results suggest that the regulation mechanism of TET2 is cell type dependent. This is reasonable for in IP-MS of TET2, we did find quite large number of interaction proteins other than β-catenin. So, more comprehensive studies are needed to unravel the mechanism of TET2 thoroughly.

In summary, this work reveals a unique mechanism of TET2 activity regulation in solid tumours. All of these results demonstrate that TET2, especially its localization and activity, is a new therapeutic intervention target for life-threatening late-stage tumours.

## Methods and materials

### Colorectal cancer samples

Colorectal cancer tissue arrays were obtained from National Engineering Center for Biochip at Shanghai (NECBS) and Taizhou Hospital of Zhejiang Province (THZP). The collection and usage of these colorectal samples were approved by the Institutional Review Boards of the NECBS and THZP in accordance with the Declaration of Helsinki. Written informed consent was obtained from the patients before the samples were collected. Paraffin sections of colorectal cancer samples were obtained from Sun Yat-sen University Cancer Center, which was approved by the Institutional Review Boards in accordance with the Declaration of Helsinki. Written informed consent was obtained from the patients before the study began. The studies with human samples were also approved by the Institutional Review Board of Guangzhou Institutes of Biomedicine and Health (No.GIBH-IRB07-2017). All the samples used in this study were collected from initial surgical treatment. Clinical information and semi-quantified IHC results were listed in Supplementary Table S1.

### Animals

All animal studies were performed in accordance with the National Institutes of Health Guide for the Care and Use of Laboratory Animals (NIH Publication No. 80-23) and were approved by the Institutional Review Board in Guangzhou Institutes of Biomedicine and Health (No.2019061). All efforts were made to minimize the number of animals used and their suffering. BALB/c^nu/nu^ mice were purchased from Beijing Vital River Laboratory Animal Technology. Mice were normally housed in groups with access to food and water ad libitum. After surgery, mice were housed individually with access to food and water ad libitum.

### Cell lines

Primary cell lines established from the colorectal cancer samples were obtained from Chi Scientific (Human Cancer PrimaCel^lTM^ 6, 2-96029) and cultured with Colorectal Tumor Cells Growth Medium as instructed. Ethics approval and informed consent were completed by Chi Scientific.

Cancer cell lines and HL7702 were obtained from Cell Bank of Chinese Academy of Sciences (http://www.cellbank.org.cn/). Human skin fibroblasts (HSFs) were obtained in previous studies [41]. Cell lines were cultured in High Glucose DMEM supplemented with 10% Fetal Bovine Serum (FBS, Excell), 1% nonessential amino acids (NEAA, Thermo Fisher), and 1% GlutaMAX (Thermo Fisher). Cells were maintained at 37 °C under 5% CO_2_.

Cell mesenchymal morphology was defined as low cell-cell interaction and high aspect ratio (over 1.5), while epithelial morphology was considered as high cell-cell interaction and low aspect ratio (below 1.5). All cells were subjected to a mycoplasma test (MycoAlert^TM^, Lonza) to ensure that they were free of mycoplasma before use. Moreover, all immortalization cell lines were subjected to STR identification.

### Tumorigenesis and tail vein injection assays

Approximately 2 × 10^6^ logarithmically growing SW620-Flag, SW620-Tet2CD, SW480- Flag and SW480-Tet2CD cells in 0.1 mL PBS were transplanted subcutaneously into right flank of 4 to 6 week-old BALB/c^nu/nu^ mice for in vivo tumorigenesis assays [42] .

Approximately 2 × 10^6^ logarithmically growing SW620-Flag, SW620-Tet2CD, SW480- Flag and SW480-Tet2CD cells in 0.1 mL PBS were injected through the tail vein for in vivo metastasis assay (n = 5). The tumor size was measured and calculated by the formula of ellipsoid (1/2 × Length × Width^2^) for tumor volume. All mice were euthanized within 6 weeks and biopsied, the subcutaneous tumors, lung and liver were removed and fixed with paraformaldehyde for subsequent paraffin embedding and staining.

### Cell-line-derived xenografts model (CDX)

SW620 cell-line-derived xenografts were established and prepared for the subsequent cohorts for drug testing. Logarithmically growing SW620 cells were transplanted subcutaneously into 4- to 6-week-old BALB/c^nu/nu^ mice for in vivo tumorigenesis. Subcutaneous tumors were surgically resected and cut into pieces (approximately 3 mm × 3 mm), one tumor piece is implanted per mouse subcutaneously into the right flank of mice. 72 mice were divided into six groups for therapy every 3 days by intraperitoneal injection (5 times in total), when CDX tumors are approximately 200 to 300 mm^3^. Vc alone (50 mg/kg), IM12 alone (1 mg/kg), high IM12 alone (5 mg/kg), combination of Vc (50 mg/kg) and low IM12 (1 mg/kg), combination of Vc (50 mg/kg) and high IM12 (5 mg/kg) were administrated as therapy groups, vehicle (5% DMSO + 40% PEG300 + 10% Tween-80 + 45% H_2_O) was used as control. Tumor volumes and mice weights were measured every 3 days. All mice were sacrificed on day 25.

### Immunohistochemistry (IHC)

The tissue was deparaffinized by placing slides into three changes of xylene and rehydrated in a graded ethanol series. The rehydrated tissue samples were rinsed in water and subjected to antigen retrieval in citrate buffer (pH 6.0). Endogenous peroxidase activity was blocked with 3% hydrogen peroxide for 15 min at room temperature, then slides were washed with PBS and incubated with the indicated antibodies overnight. The slides were then washed with PBS and incubated with secondary antibody in universal two-step detection kit (ZSGB-BIO, PV9000) according to the instruction of the manufactures. The images of tissue microarrays were scanned by 3D HISTECH Digital slice scanner.

The levels of TET2, 5hmC, and 5mC were semi-quantitatively scored in a blinded fashion based on staining intensity and frequency of positive cells of tumor tissue. Two pathologists who were blinded to clinical parameters individually reviewed and scored the stained tissue sections. The staining intensity was scored as 0 (negative), 1 (weak), 2 (medium) and 3 (strong), and the percentage of positively stained cells was defined on a scale of 0–3 (0: 0-5%, 1: 6–25%, 2: 26–50% and 3: >50%). Then multiply the two scores, and the final staining scores of 0-3 or 4–9 were considered as low or high expression levels, respectively. TET2 nuclear localization is decided with a significant higher level of TET2 in cell nuclear and TET2 cytoplasm localization is decided with a significant higher level of TET2 in cell cytoplasm.

### Gene knockout and lentivirus infection

Cells were transfected with virus produced from pLentiCRISPRv2 system with the appropriate gRNA sequences designed on a website (crispr.mit.edu) and selected with puromycin (Gibco, A1113803). Cells were plated into 96 well plates in concentration at approximately 1 cell/well. Genomic DNA was isolated from surviving cells and the knockout of target gene was verified. The homozygous colonies with unimodal sequencing results (with frame shift mutation) were used. All gRNA sequences were listed in Supplementary Table S7.

Lentivirus was packaged by co-transfection of vectors encoding target genes and the packaging plasmids pMD2.G and psPAX2 into HEK293T cells. Viruses were collected and used to infect cell lines. Puromycin was used to select cell lines with genomic incorporation and stable expression of the target genes.

### Immunoblotting (IB), Immunoprecipitation (IP) and Mass Spectrum (MS)

Whole cell extracts were obtained with lysis buffer (50 mM Tris pH 8.0, 150 mM NaCl, 10% Glycerol, 0.5% NP40, 1 mM Phenylmethanesulfonyl fluoride and freshly added protease inhibitor cocktail). After centrifugation at 13,000 rpm for 15 min at 4 °C, soluble proteins were quantified by BCA assay kit.

Approximately 100 μg of cell lysates were used for immunoblotting. Samples were loaded onto SDS-PAGE and transferred to PVDF membrane. The membrane was washed with TBST buffer and incubated with indicated antibodies. Minichem Biosystem (SAGECREATION) were used to semi-quantify the target proteins.

Approximately 1 mg of cell lysates were incubated with indicated antibody (normally Anti-Flag, Sigma) overnight at 4 °C on a rotation wheel. Combined Protein A/G magnetic beads (Bio-rad, 1614833) were added for another 1.5 hours. Elution was performed as instructed and samples were then subjected for immunoblotting.

For IP-MS, precipitated proteins were eluted as reported previously [43]. Briefly, 100 μl of elution buffer (2 M urea, 10 mM DTT and 50 mM Tris pH 8.5) for 20 min, 50 mM iodoacetamide (Sigma, I1149) for 10 min in dark, and 250 ng of trypsin (Promega, V5280) for 2 hours were used to treat the beads. The supernatant was collected with 100 μl of elution buffer for another round of 5 min incubation. Combined elutes were digested with 200 ng of trypsin overnight at RT, acidified to pH < 2 by adding approximately 10 μl of 10% Formic acid (Sigma, 1002641000) and desalted using C18 Stagetips (Sigma, 66883-U) prior to MS analyses. Each experiment was performed in technical triplicate.

MS analysis was performed as reported previously [44]. Tryptic peptides were separated using a 140 min of total data collection with an Easy-nLC 1200 connected online to a Fusion Lumos mass spectrometer (Thermo Fisher). Scans were collected in data-dependent top-speed mode with dynamic exclusion at 90 sec. Raw data were analyzed using MaxQuant version 1.6.0.1 search against Mouse Fasta database, with label free quantification and match between runs functions enabled. The output protein list was analyzed and visualized using DEP package.

### Immunofluorescence (IF)

Cells were fixed with 4% paraformaldehyde after removing the medium and washing three times with PBS. The samples were washed twice with PBS and blocked with blocking buffer (PBS containing 10% normal goat serum (Beyotime, C0265), 1% bovine serum albumin (Genview, FA016) and 0.3% Triton X-100). Antibodies were diluted with blocking buffer and incubated with the samples for 12 hours at 4 °C or 2 hours at RT. Three PBS washes were performed after each time of antibody incubation. Immunofluorescence was detected with a Zeiss LSM800.

### Cytoplasm and nuclear protein fractionation

Cells used for cytoplasm and nuclear protein fractionation were washed in cold PBS and counted. Cytoplasm and nuclear protein fractions from 500*10^4^ cells were harvested as the instruction of Subcellular Protein Fractionation Kit for Cultured Cells (Thermo Fisher Scientific, 78840). Proteins were then separated by SDS-PAGE and electrotransferred to PVDF membranes. Following primary and secondary antibody incubation, signals of target proteins were analyzed with Minichem Biosystem.

### Quantitative PCR (qPCR)

Total RNA was extracted from the cells using TRIzol (Thermo Fisher, 15596018), and 2 μg of RNA were used to synthesize cDNA with ReverTra Ace® (Toyobo, TRT-101) and oligo-dT (Takara) according to the manufacturers’ instructions. The transcript levels of the genes were determined using Vazyme 2*Master Mix (Vazyme, Q311-02-AA,) and a CFX-96 Real-Time system (CFX96, Bio-Rad).

### Wound healing assay

Cells were seeded onto a 6-well plate and grown to 90% confluence. Confluent cells were then scratched using a 200 μl pipette tip. After rinsing three times with PBS to remove detached cells, the remaining cells were incubated in serum-free medium (DMEM medium, HyClone) at 37 °C for 96 hours. The wound status was recorded (Olympus Corporation, Tokyo, Japan) at hour 0, 24, 48, 72 and 96 after scratching. Scratch covered by the migrated cells were quantified via ImageJ software (version 1.48; National Institutes of Health, Bethesda, MD, USA).

### Colony forming assay

For clone formation assay, cells were plated in 12-well plates at a concentration of 800 cells/well and incubated for 8 days. Then cells were washed with PBS for three times, and fixed with 70% ethyl alcohol, stained with Crystal Violet and dried. Plates were scanned with the aid of a standard scanner system (MFC-7340, BROTHER). The corresponding treatments in different cells are shown in the figure, and the colony sizes were quantified via Image J software.

### Sodium bisulfite sequencing

Genomic DNA was extracted using a Wizard Genomic DNA Purification Kit (Promega A1125) according to the manufacturer’s instructions. The purity of the DNA was determined using a K5500 spectrophotometer, and DNA quantification was performed using a Qubit® 3.0 fluorometer.

Genomic DNA (200 ng) was exposed overnight to a mixture of 40.5% sodium bisulfite and 10 mM hydroquinone. Subsequently, regions of the target promoters were amplified by PCR. The PCR products were cloned into the pMD18-T vector (Takara), propagated in DH5α, sequenced, and mapped to genome.

### Dot-blotting

Genomic DNA (1.2 ug/uL, 0.8 μL, 2 fold dilution to have six spots) was pipetted onto the nitrocellulose membrane. After drying, 1 min ultraviolet crosslinking, and 1 hour incubation with 5% milk, antibodies against 5mC or 5hmC were incubated for 2 hours at RT. The membrane was washed in PBS containing 0.1% Tween (PBST) five times. Then the membrane was incubated with secondary antibodies at a dilution of 1:2000 for 1 hour at RT. The membrane was washed again with PBST for five times. Finally, DNA on the membrane was visualized using enhanced chemiluminescence reagents (Millipore, WBKLS0500).

### DNA demethylation assay with HPLC or LC-MS/MS

For global DNA methylation level, DNA was extracted using a Wizard Genomic DNA Purification Kit (Promega) according to the manufacturer’s instructions. Purified DNA with QIAquick Nucleotide Removal kit was digested with nuclease P1, phosphodiesterane I and alkaline phosphatase [45]. The resulting DNA digestion solution was analyzed by electrophoresis on a 1% agarose gel to verify complete digestion. The DNA digestion solution was diluted 3-fold, ultrafiltrated to remove molecules larger than 3k dalton.

For in vitro DNA demethylation assay, the CpG site in the middle of the two oligonucleotides were methylated CpG sites. Annealed oligonucleotides (normally 1 μM, but different when indicated) were incubated with cell lysis in the presence of 1 mM α-ketoglutaric acid (Sigma), 100 μM Fe(NH_4_)_2_(SO_4_)_2_ (Sigma), 1 μM ATP (Sigma), 1 mM ascorbic acid, and HEPES buffer (pH 8.0). The reactions were carried out at 37 °C for 6 hours.

For 5mC quantification, each sample (10 μl) was loaded and analyzed on Agilent 1260 BIO with a C18 reverse-phase column (2.1 × 50 mm, 1.8 μm, Agilent ZORBAX Eclipse Plus C18). The mobile phase was 7 mM ammonium acetate (pH 6.7) in water and methanol (v/v, 19:1); the flow rate was 0.3 ml per min, and the detector was set at 280 nm.

### RNA-seq

RNA was extracted from cells using TRIzol reagent. RNA-Seq libraries were prepared for each RNA sample using the TruSeq RNA Sample Preparation Kit v2 (RS-122-2001, Illumina). The sequencing was done using a NextSeq 500 High Output Kit v2 (75 cycles) (FC-404-1005, Illumina) according to the manufacturer’s instructions. The depths of sequencing are 10 M pair-end reads of length 50NT. The raw data obtained using RNA-seq were tested for basic quality control with the FASTQC tool, and the filtered reads were pre-processed using the TRIMMOMATIC tool (PE.fa:2:30:10:8:true LEADING:3 TRAILING:3 SLIDINGWINDOW: 4:15 MINLEN:36). The percentage of qualified reads was 97.37%. Reads were aligned to a transcriptome index generated from the Ensembl annotations (v67), using RSEM, bowtie2, and sequencing data using GC-content normalization [46]. Gene Oncology analysis was performed by using DAVID 6.8 (https://david-d.ncifcrf.gov/) [47], and STRING 11 analysis was performed on https://string-db.org/ [48].

### Whole gerome bisulfate sequencing

The purified genomic DNA was shipped on dry ice to Annoroad Gene Technology Co. Inc., Beijing, China for WGBS. Clean reads were mapped to the mouse genome (GRCm38/mm10) using previously reported methods and software [49]. The raw sequencing data, clean reads, and methylation information on each cytosine were provided for further analysis.

### Materials

Additional materials used in the current studies were listed in Supplementary Table S7.

### Statistical analysis

All experiments were repeated for at least five times (*n* ≥ 5) except sequencing experiments. The criterion for samples exclusion was pre-established. If the values obtained were more than twice the SEM of the mean, we excluded the samples. The normalization step eliminates the batch difference by hypothesizing that the performance of control group was consistent. For animal studies, each group included at least 5 mice. Samples/organisms/participants were allocated into experiments groups with simple randomization. Investigators were not blinded to group allocation in some experiments with cell lines, but multiple investigators repeated these experiments with no knowledge of the results. The data were analyzed and compared using two-tailed student t-test, one-way ANOVA, or two-way ANOVA with multiple comparisons in GraphPad Prism 7.0. Error bars and “n” represent the standard deviation (standard error only if indicated) and the number of independent experiments, respectively. “*”, “**”, and “***” represent significant differences (*P* < 0.05, *P* < 0.01, and *P* < 0.001, respectively) from the indicated control groups. Detailed information was listed in Supplementary Table S8.

## Supplementary information

Supplementary Information

Figure S1

Figure S2

Figure S3

Figure S4

Figure S5

Figure S6

Table S1

Table S2

Table S3

Table S4

Table S5

Table S6

Table S7

Table S8

original western blots

aj-checklist

## Data Availability

The RNA-seq results generated during this study are available at Gene Expression Ominibus (GEO) under accession number (GSE101386 & GSE154646). The WGBS results generated during this study are available at GEO under accession number (GSE154734). The IP-MS results generated during this study are available at ProteomeXchange Consortium via the PRIDE partner repository with the dataset identifier PXD020080 (Reviewer Account, Username: reviewer51361@ebi.ac.uk, Password: SwbhCoEB). The details and corresponding accession number were listed in Supplementary Table S7. This study did not generate code.
