# Supplementary material for: Nuclear localization of TET2 requires β-catenin activation and correlates with favourable prognosis in colorectal cancer
Source: Cell Death Dis. 2023 Aug 24;14(8):552. doi: 10.1038/s41419-023-06038-x (PMC10449923; doi:10.1038/s41419-023-06038-x)
Supplement: Supplementary file 1 — Supplementary Information [file 41419_2023_6038_MOESM1_ESM.docx]

**Supplementary Information for:**

**Nucleocytoplasmic Shuttling of TET2 Regulates Colorectal Cancer Progression**

Changpeng Li^1,2,#,*^, Jingcai He^1,3,#^, Fei Meng^1,2,4,5,#^, Fuhui Wang^1,4,#^, Hao Sun^1,2^, Huizhong Zhang^6^, Linna Dong^1,4^, Mengdan Zhang^1,4^, Qiaoran Xu^1,4^, Lining Liang^1,2^, Yuan Li^1^, Tingting Yang^1,2,4^, Meiai He^1,4^, Tao Wang^1,4^, Jiechun Lin^1,7^, Jiaqi Sun^8^, Qiuling Huang^1^, Lin Guo^1,2^, Xiaofei Zhang^1^, Shijuan Mai^6,*^, Hui Zheng^1,2,4,7,*^

**Supplementary Figure Titles and Legends**

**Supplementary Figure S1**

**High TET2 in nucleus favors better survival.**

(A) Representative IHC staining for TET2 in CRC samples.

(B-D) Kaplan–Meier survival plots of the two categories of 380 CRC samples are presented (B). The two cohorts were also separately analyzed in (C) and (D).

(E) High TET2 in nucleus was associated with high levels of 5hmC.

(F-G) CRC samples were classified into three categories, low TET2, high TET2 in nucleus and high TET2 in cytosol. Survival analyses were performed with samples from Guangzhou cohort (F) and Shanghai-Taizhou cohort (G).

(H) High TET2 in nucleus was associated with low Dukes’ stages.

(I) Kaplan–Meier survival plots of the two categories of 72 CRC samples with TET2 low expression are presented. Additional statistic information was provided Supplementary Table S8.

**Supplementary Figure S2**

**TET2 inhibited the growth and migration of SW480 cells but not SW620 cells.**

(A-E) Tet1CD, Tet2CD, or Tet3CD was overexpressed in SW480 and SW620 cells (A) and the performance of these cells in colony forming assay were determined (B-C). The inhibition of growth was measured with CCK8 (D-E). Inhibition effect was only observed in SW480 expressing Tet2CD.

(F-K) Tet2FL was overexpressed (F-G). The proliferation of these cells in colony forming assay was determined (H). Cell growth was measured with CCK8 (J-K). Tet2FL inhibited the proliferation of SW480 but not SW620 cells.

(L-Q) *TET2* was knocked out in SW480 and SW620 cells with CRISPR-Cas9 system (L). Colony forming (M-N) and wound healing assays (O) of these cells were determined. CCK8 analysis was carried out with indicated cells (P-Q). *TET2* knockout facilitated the proliferation of SW480 cells but not SW620 cells.

All experiments were repeated for at least 5 times (n≥5). The S.D. was shown. Additional statistic information was provided Supplementary Table S8.

**Supplementary Figure S3**

***RORA-SPARC* axis is essential for the cancer suppression induced by TET2.**

(A) Demethylation on the promoters of RORA and SPARC was determined with bisulfite sequencing in indicated tissues.

(B) *RORA* and *SPARC* were knocked out in SW480 and SW620 cells with CRISPR-Cas9 system.

(C) The abilities of Tet2CD overexpression to affect the performance of these cells were valued via colony forming assay.

(D) The abilities of Tet2CD overexpression to affect the migration of these cells were valued via wound healing assays.

All experiments were repeated for at least 5 times (n≥5). The S.D. was shown. Additional statistic information was provided Supplementary Table S8.

**Supplementary Figure S4**

**β-catenin is a crucial protein in regulating Tet2 location.**

(A-B) Proteomic analyses were employed to detect any protein that may bind TET2, 148 and 127 proteins were revealed to interact with TET2 in SW480 and SW620 cells respectively.

(C) The interaction between β-catenin and TET2 was confirmed by IP in SW480 and SW620.

(D-E) RNA-seq analysis revealed the differences in gene expression between SW480 and SW620 **(D)**. Genes related to canonical WNT-β-catenin pathway and fatty acid metabolism were enriched in SW480 and SW620 (E), respectively

(F-H) *CTNNB1* was knocked out in SW480 cells with CRISPR-Cas9 system (F). The ability of Tet2CD to affect *CTNNB1*^-/-^ SW480 cells in colony forming (G) was summarized. The ability of Tet2CD was also summarized in SW620 cells expressing *CTNNB1* or *CTNNB1mu* (H).

(I-J) The DNA demethylation abilities of nuclear extraction (I) and whole cell lysis (J) of cells expressing Tet2CD or Tet2CD-mt were determined in a biochemistry assay *in vivo.*

(K) Cytoplasm and nuclear protein fractionation analysis of indicated cells.

(L) Overexpression of Tet2CD-mt did not induce tumor growth prohibition (L).

All experiments were repeated for at least 5 times (n≥5) except sequencing experiments. The S.D. was shown in (F-N). Additional statistic information was provided Supplementary Table S8.

**Supplementary Figure S5**

**EMT regulates the localization of TET2.**

(A-B) TGFβ (TGFβ1/2/3, 1 ng/mL each) or RepSox (1 μM) were used to treat cells for three days before RNA-seq. The successful EMT or MET was confirmed by using a previously reported scoring system for epithelial and mesenchymal states.

(C) Cell migration was measured with live imaging in indicated cells.

(D-F) The nuclear localization of overexpressed Tet2CD treated with TGFβ or RepSox in SW480 cells was determined with immunofluorescence (D), and the global DNA methylation levels were determined with dot blot (E) and colony forming assays F) were performed.

(G-I) Experiments were performed with SW620 cells as in (D-F)

All experiments were repeated for at least 5 times (n≥5) except sequencing experiments. Additional statistic information was provided Supplementary Table S8.

**Supplementary Figure S6**

**β-catenin is required for TET 2 to regulate DNA methylation and cell growth**

(A-B) Compounds (the β-catenin inhibitor, IWR1, 25 μM) were used to treat SW480 cells expressing Flag or Tet2CD. The proliferation of cells (A), and the activation of RORA and SPARC were determined (B).

(C) Cytoplasm and nuclear protein fractionation analysis of indicated cells treated with IWR1 and IM12.

(D-E) Compounds (the β-catenin activator, IM12, 3.8 μΜ) were used to treat SW620 cells expressing Flag or Tet2CD. The proliferation of cells (D), and the activation of RORA and SPARC were determined (E).

(F-G) Generalization of different categories of cell lines based on their response to the combination of Vc and IM12.

All experiments were repeated for at least 5 times (n≥5). The S.D. was shown. Additional statistic information was provided Supplementary Table S8.

**Supplementary Table Legends**

**Supplementary Table S1**

**Clinical information and IHC results of 380 colorectal cancer samples**

Clinical information of the current colorectal cancer samples were obtained from THZP, and Sun Yat-sen University Cancer Center. Personal information was not provided. The level of TET2 (low or high), localization of TET2 (low, high in nucleus, and high in cytosol), level of 5mC (low or high), and level of 5hmC (low or high) were determined with IHC and listed.

**Supplementary Table S2**

**High TET2 in the nuclei correlates with several clinicopathological characteristics**

Relationship between TET2 and general clinicopathological characteristics of CRC patients from THZP and Sun Yat-sen University Cancer Center cohorts; and multi-variant analysis of all patients in this study.

**Supplementary Table S3**

**Information and functional studies results of cancer cell lines**

Brief information of the cell lines used in the current study was provided. The abilities of Tet2CD overexpression to regulate cell growth in a colony formation assay, global DNA methylation level, the expression of RORA and SPARC were summarized.

**Supplementary Table S4**

**Collected analysis of WGBS and RNA-seq of SW480 and SW620 cells**

12715 protein-coding genes with significant expression in at least one sample during the current RNA-seq analysis were summarized. Genes with larger upregulation in SW480 or in SW620 cells were labelled in column M. 285 further selected genes were labelled in column N. Additional analysis on these 285 genes were listed in column Y to AB.

The average methylation levels of all qualified CpG sites around TSS (-2.0 ~+2.0 kb) were considered as the methylation levels of corresponding genes. The results for 12715 protein-coding genes were listed in column S to X.

**Supplementary Table S5**

**IP-MS results of Tet2CD in SW480 and SW620 cells**

Tet2CD was overexpressed in SW480 and SW620 cells. Tet2CD was immunoprecipitated and subjected for MS analysis.

**Supplementary Table S6**

**RNA-seq results of SW480 and SW620 cells with TGFβ or RepSox treatment**

SW480 and SW620 cells with/without Tet2CD overexpression were treated with TGFβ (TGFβ1/2/3, 1 ng/ml each) or RepSox (1 μM) for three days. RNA-seq were then performed. The expressions of 15969 genes with significant expression in at least one sample during the current RNA-seq analysis were summarized.

**Supplementary Table S7**

**Material used in the current study**

As Title.

**Supplementary Table S8**

**Additional Statistic information**

As Title.
