# Supplementary figures and images for: Nuclear localization of TET2 requires β-catenin activation and correlates with favourable prognosis in colorectal cancer
Source: Cell Death Dis. 2023 Aug 24;14(8):552. doi: 10.1038/s41419-023-06038-x (PMC10449923; doi:10.1038/s41419-023-06038-x)

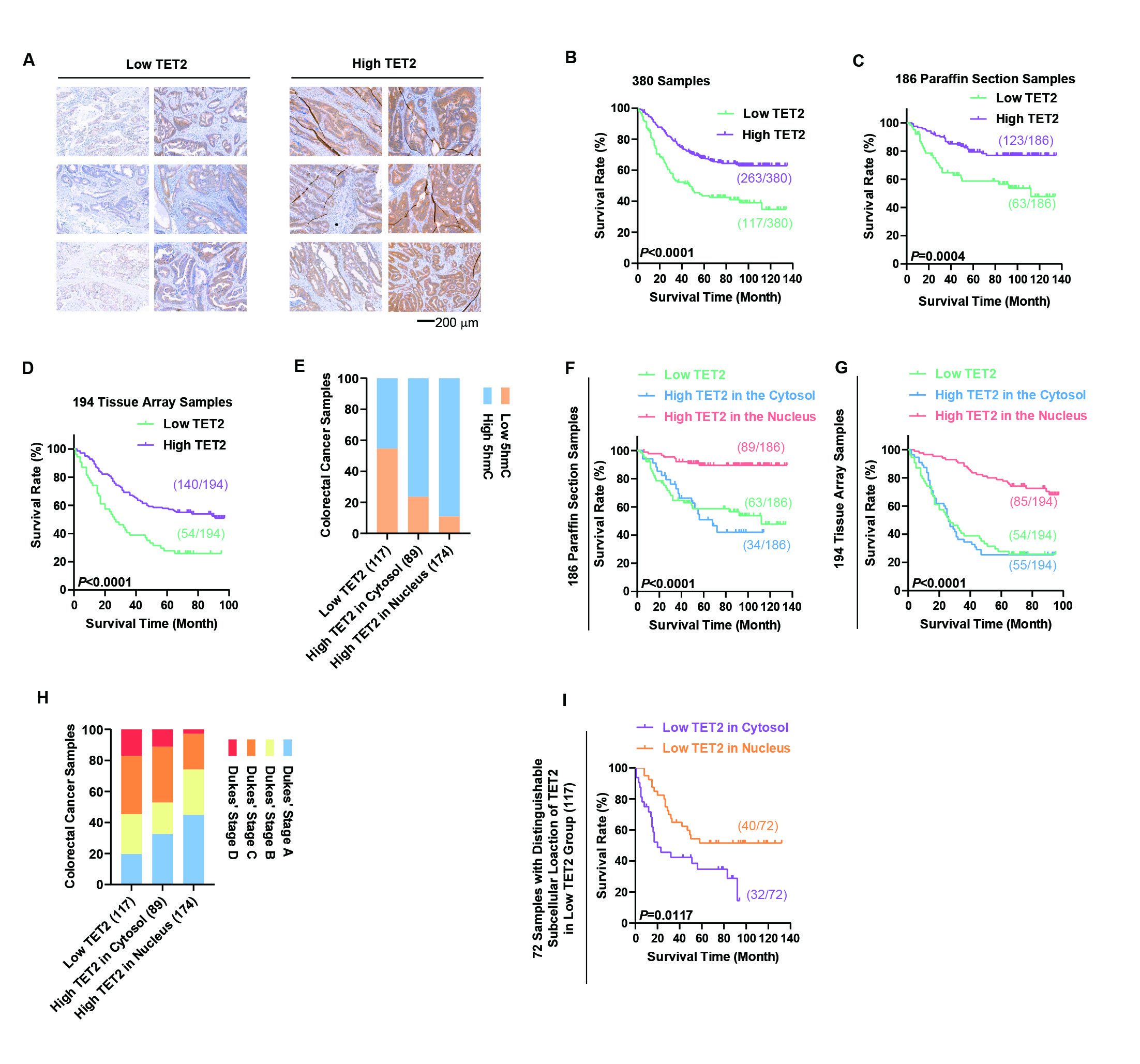

Supplement: Supplementary file 2 — Figure S1 [file 41419_2023_6038_MOESM2_ESM.jpg]

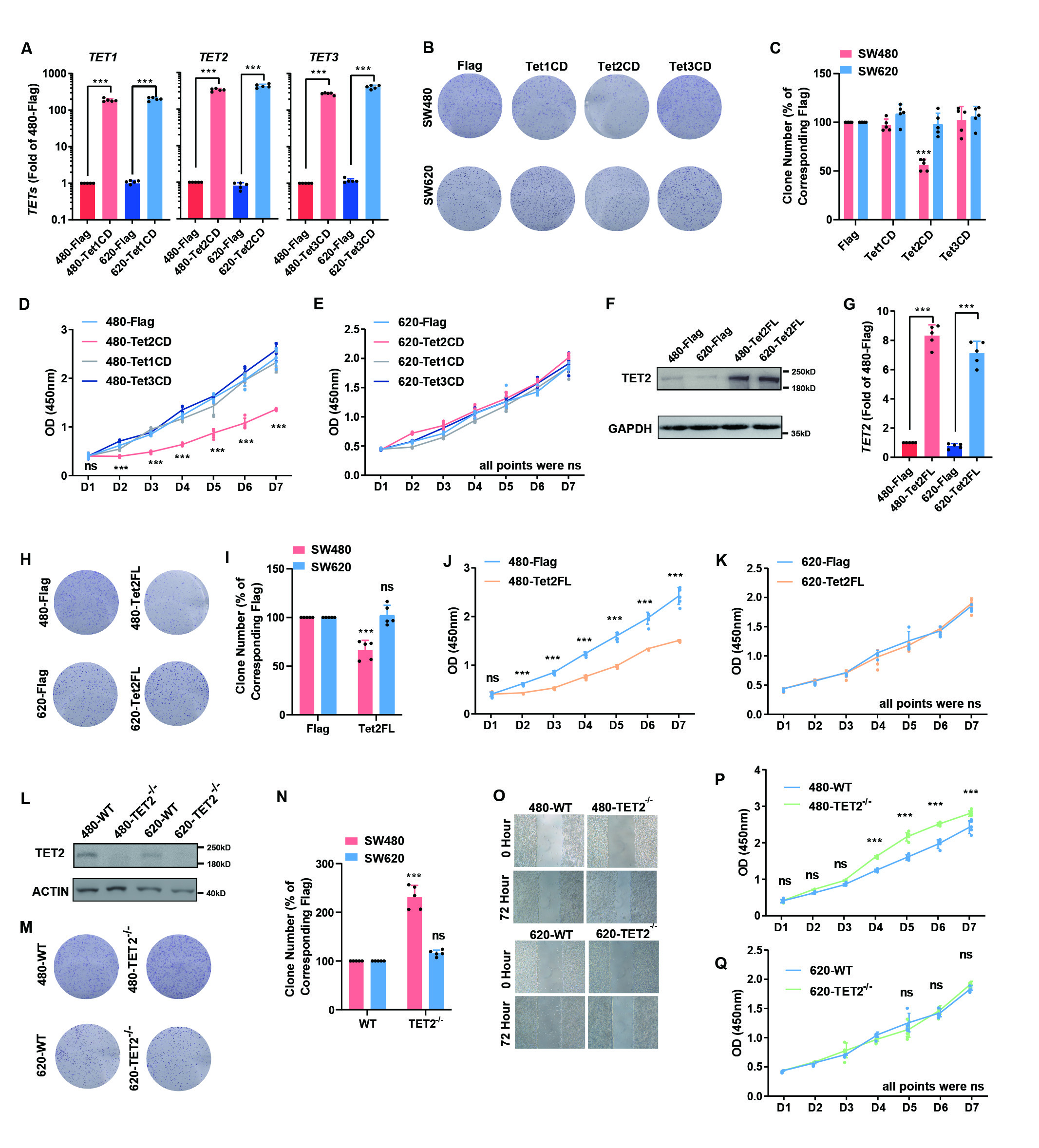

Supplement: Supplementary file 3 — Figure S2 [file 41419_2023_6038_MOESM3_ESM.jpg]

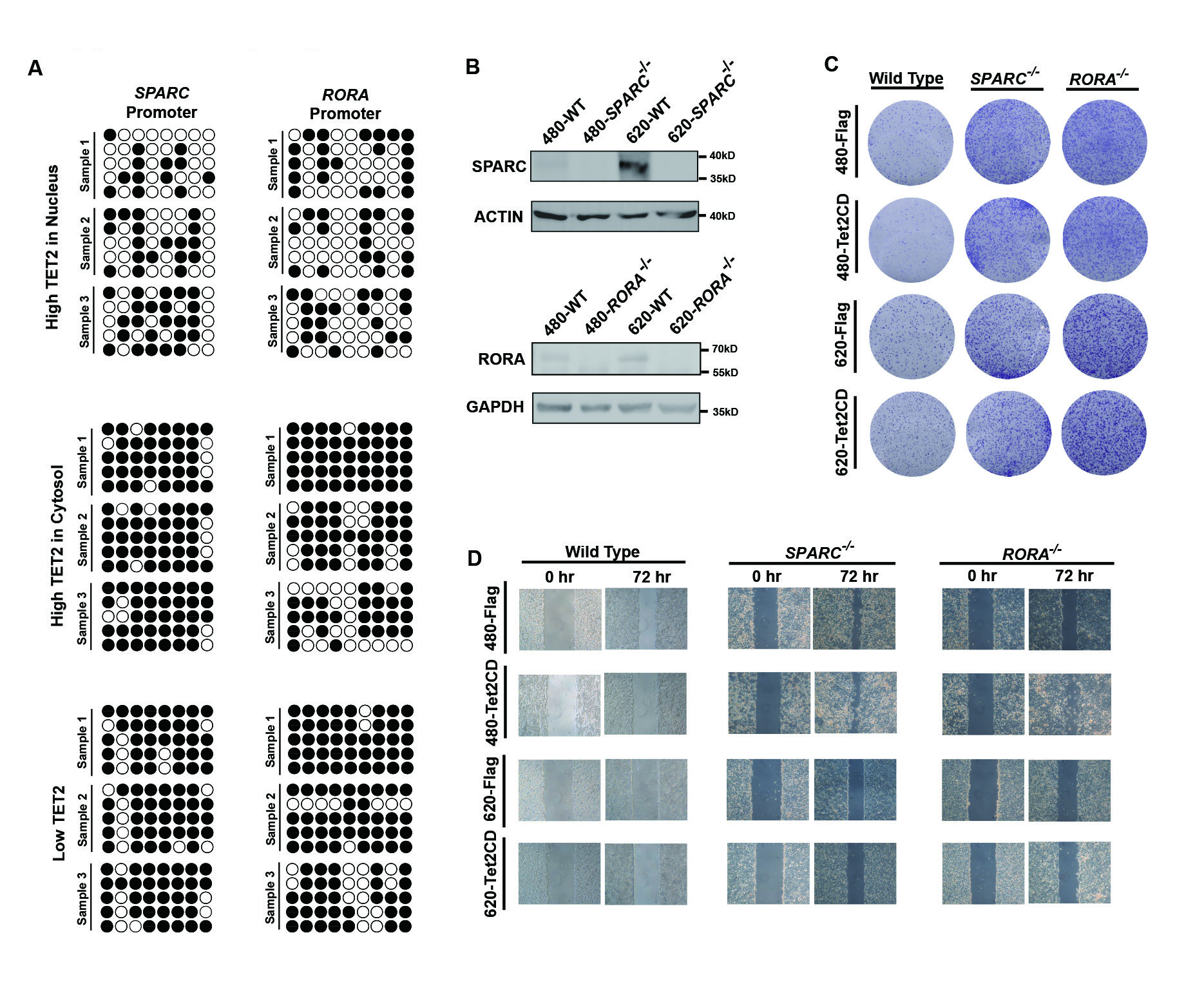

Supplement: Supplementary file 4 — Figure S3 [file 41419_2023_6038_MOESM4_ESM.jpg]

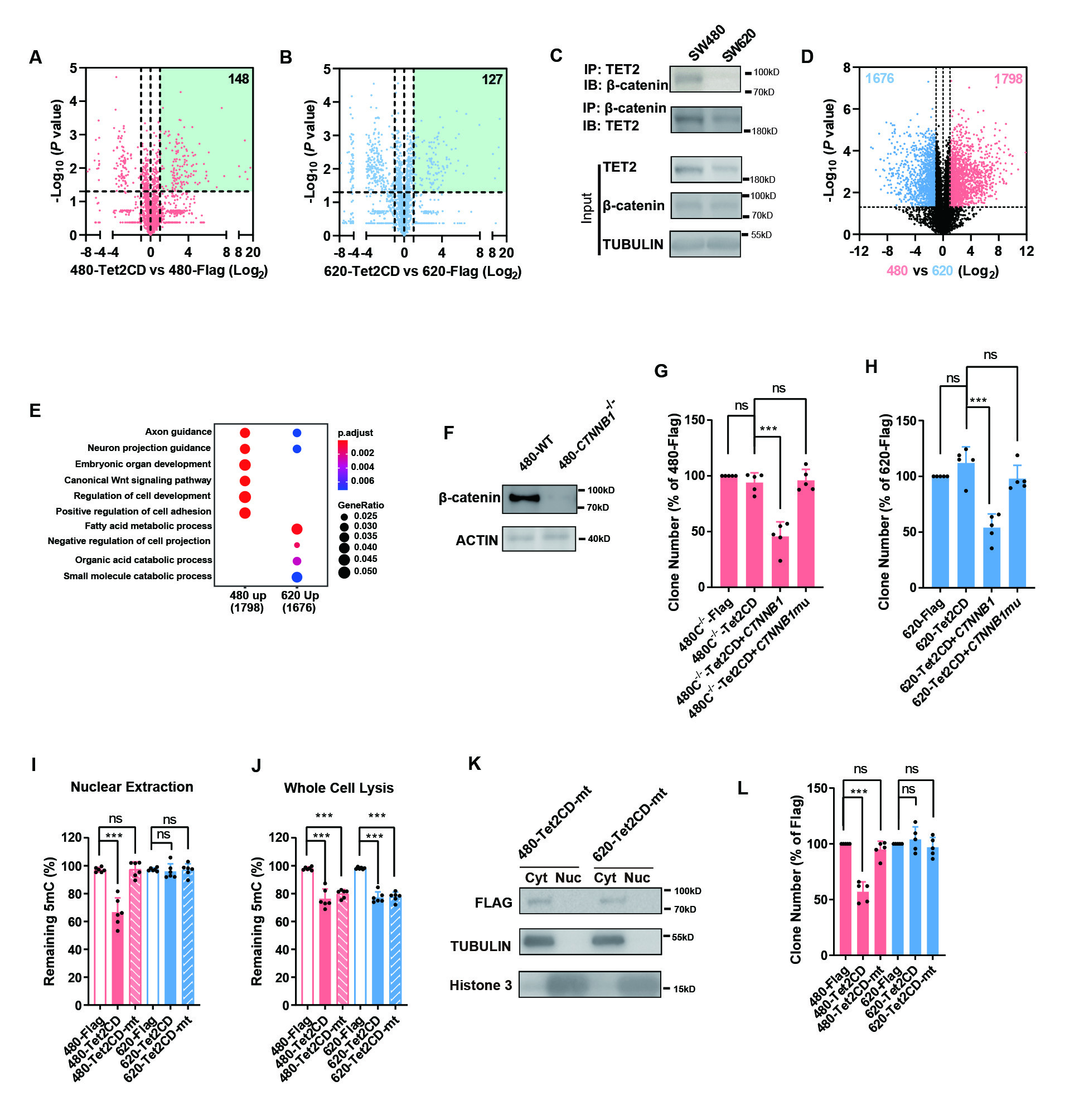

Supplement: Supplementary file 5 — Figure S4 [file 41419_2023_6038_MOESM5_ESM.jpg]

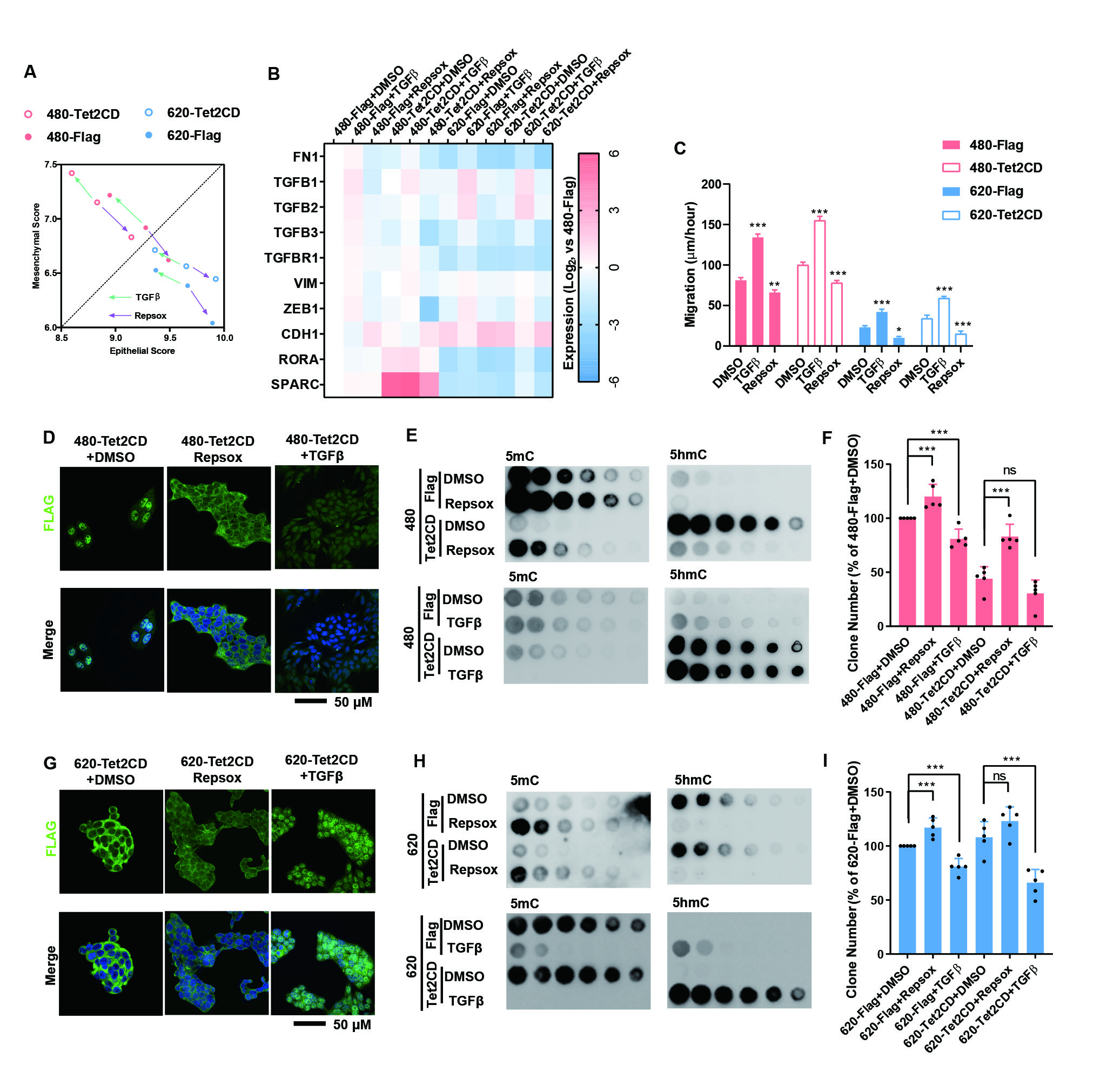

Supplement: Supplementary file 6 — Figure S5 [file 41419_2023_6038_MOESM6_ESM.jpg]

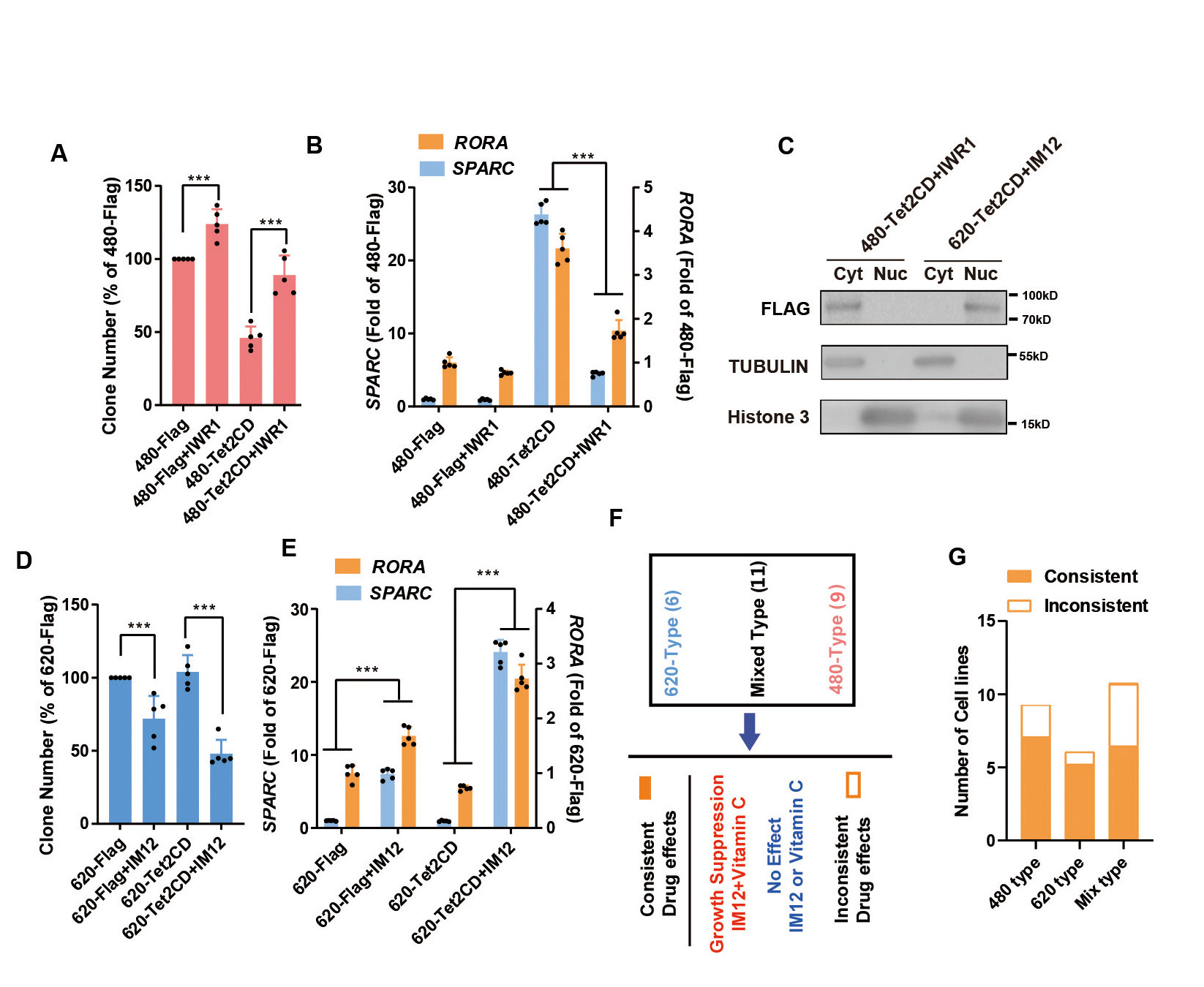

Supplement: Supplementary file 7 — Figure S6 [file 41419_2023_6038_MOESM7_ESM.jpg]
