# Supplementary material for: Nuclear localization of TET2 requires β-catenin activation and correlates with favourable prognosis in colorectal cancer
Source: Cell Death Dis. 2023 Aug 24;14(8):552. doi: 10.1038/s41419-023-06038-x (PMC10449923; doi:10.1038/s41419-023-06038-x)

Figure 2C

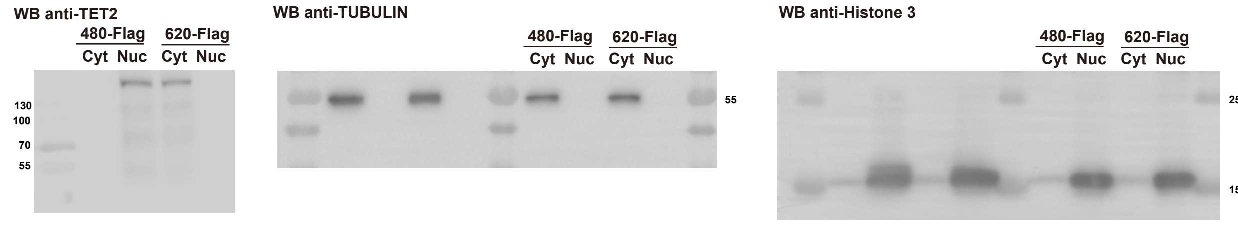

Figure3 D

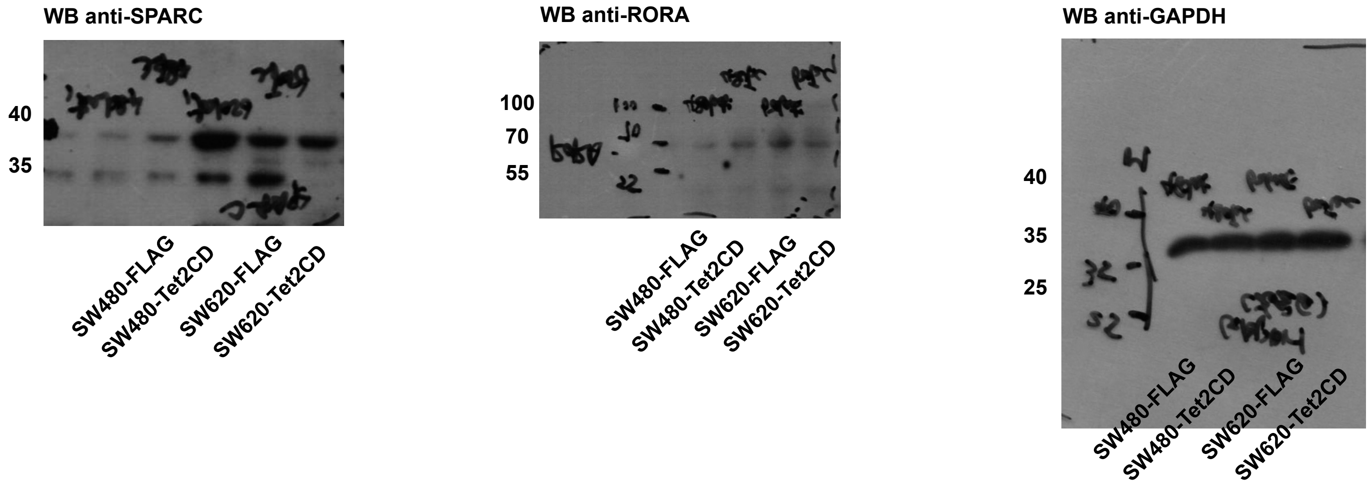

Figure4 C

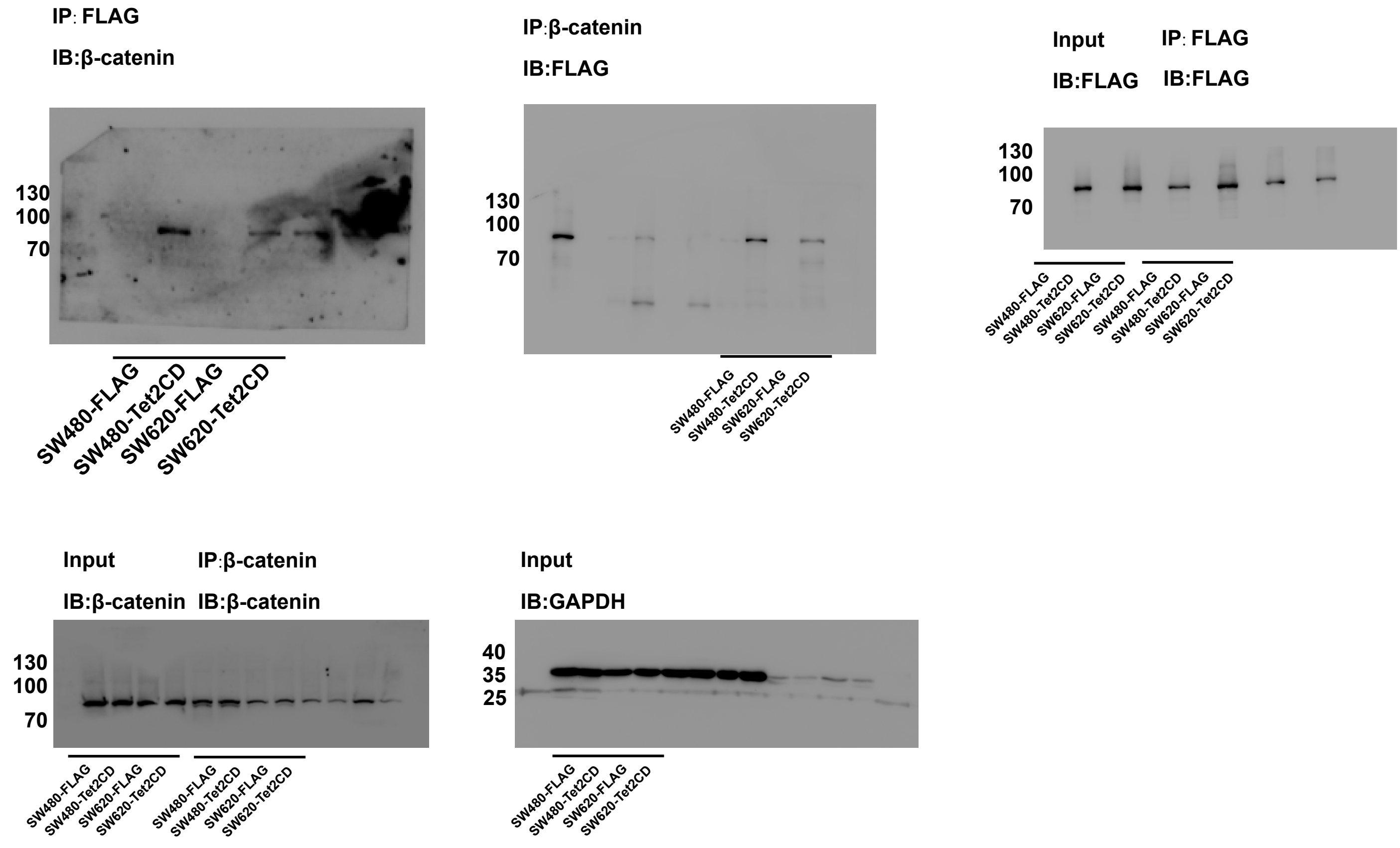

Figure 4F

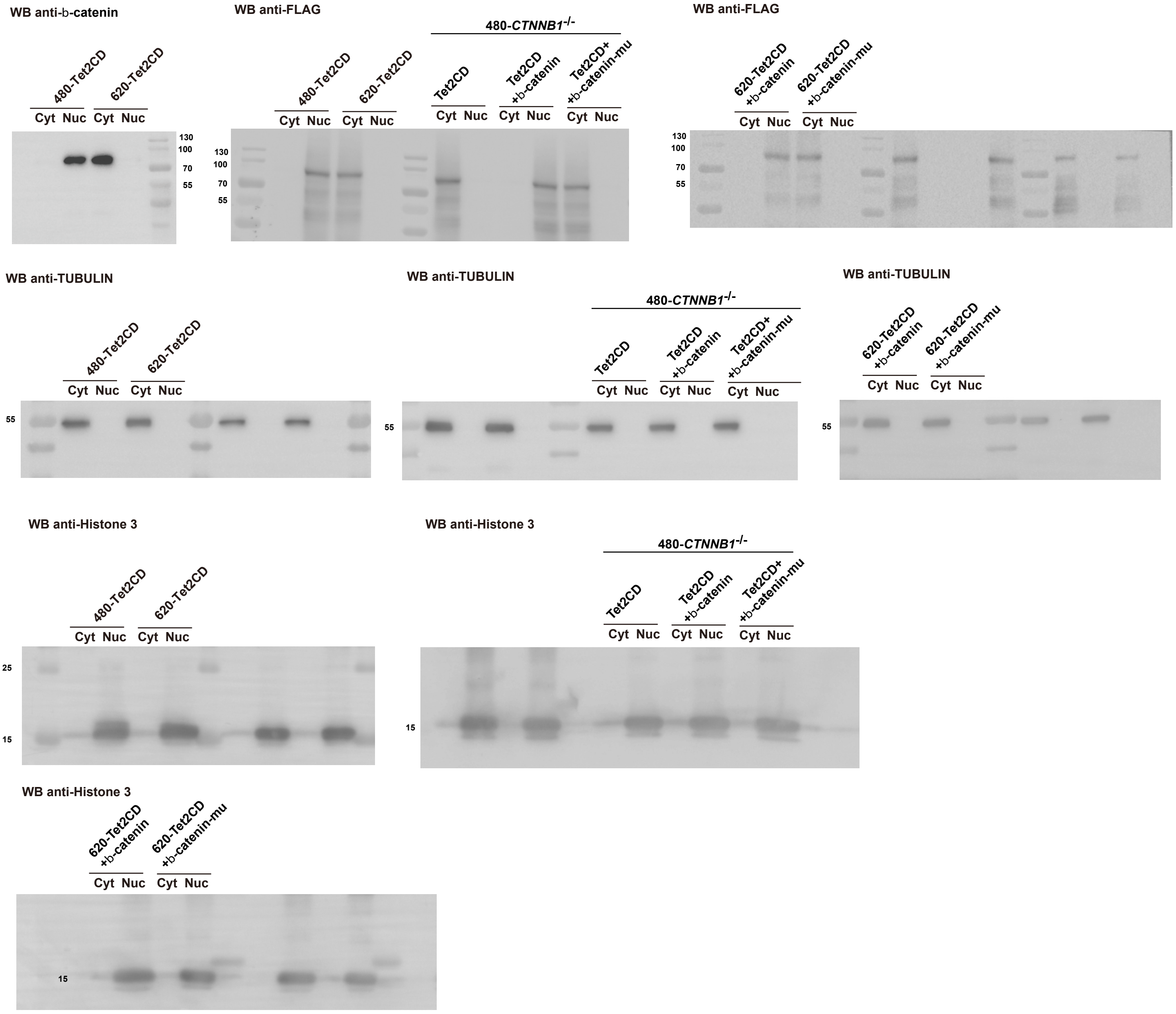

Figure5 A

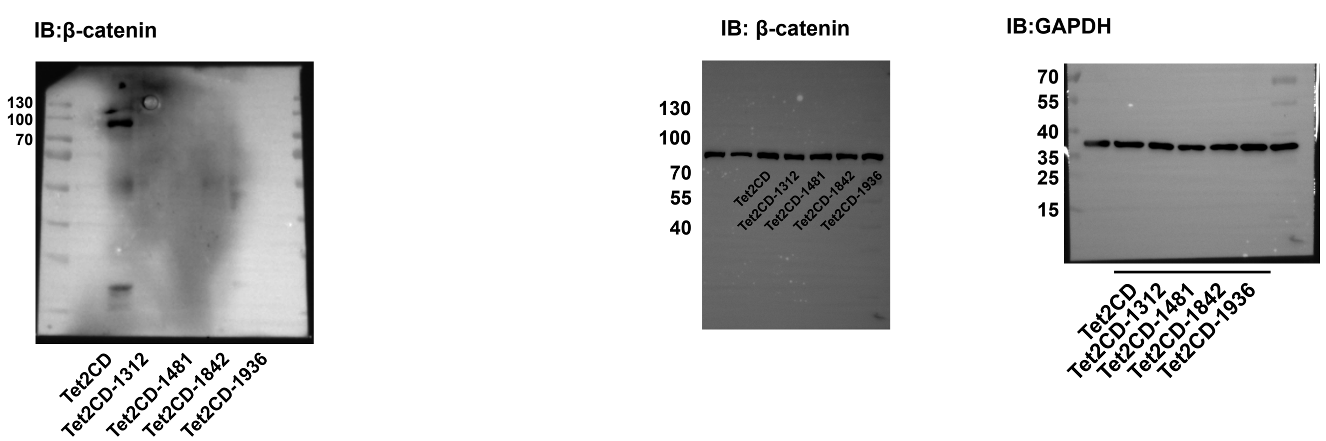

Input

IB:FLAG

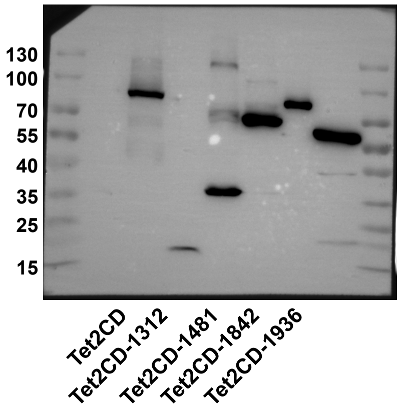

Figure5 B

IP: FLAG

IB:β-catenin

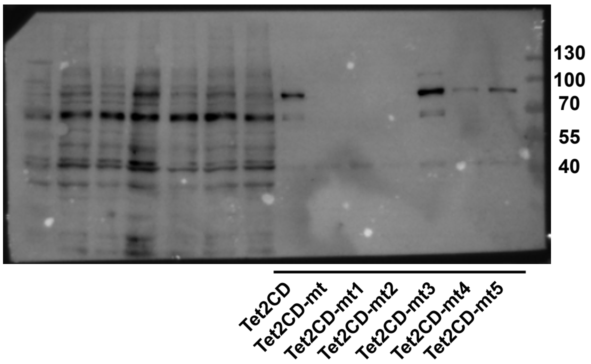

Input

IB:FLAG

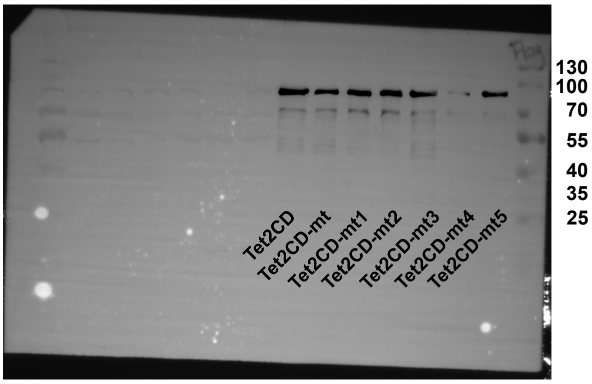

Input

IB:β-catenin

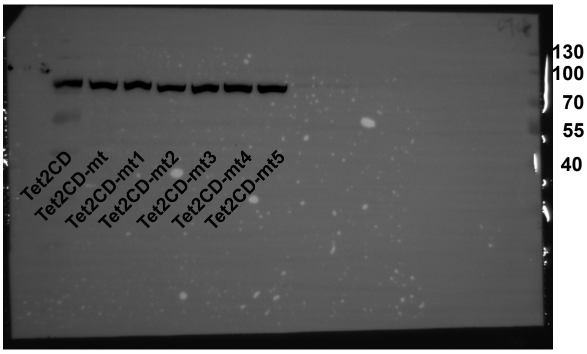

IP: FLAG

IB:β-catenin

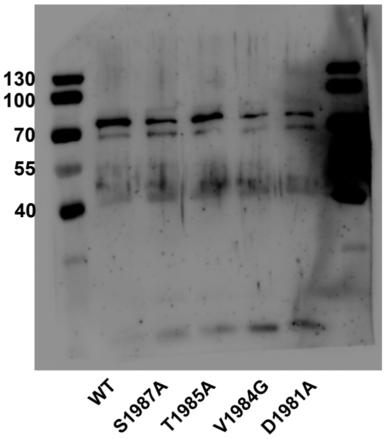

Input

IB:FLAG

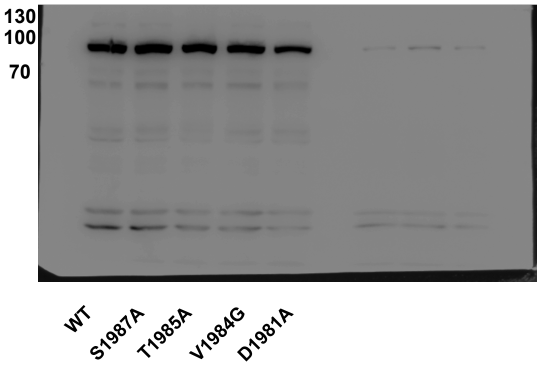

Input

IB:β-catenin

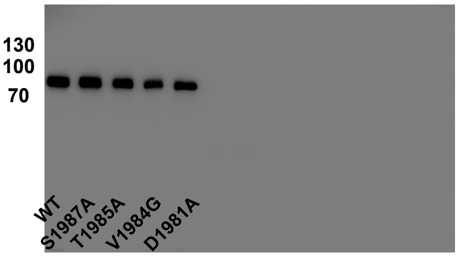

Figure S2F

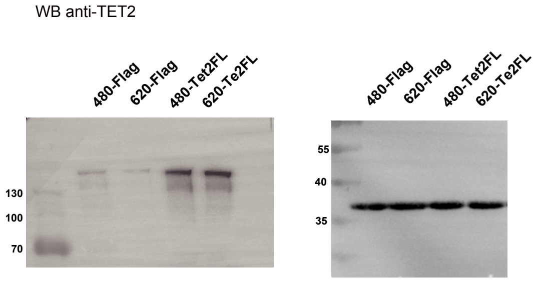

Figure S2L

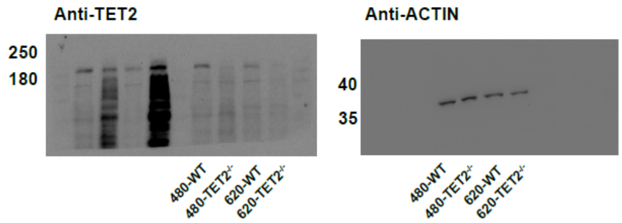

FigureS3 B

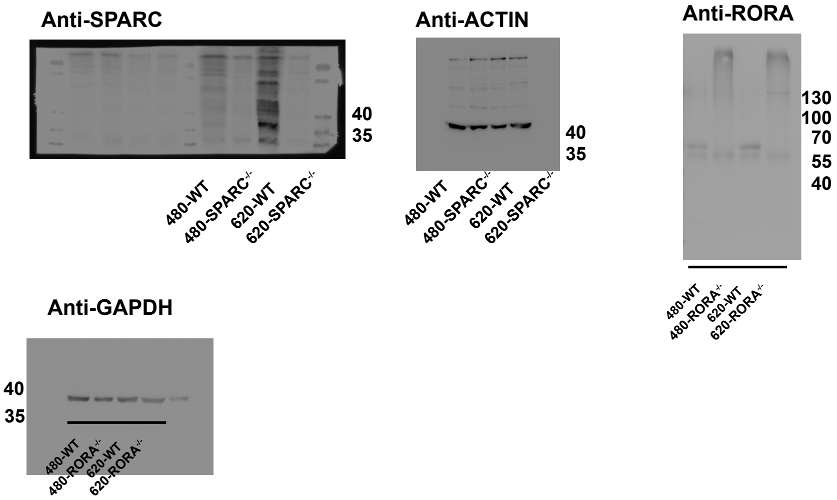

Figure S4C

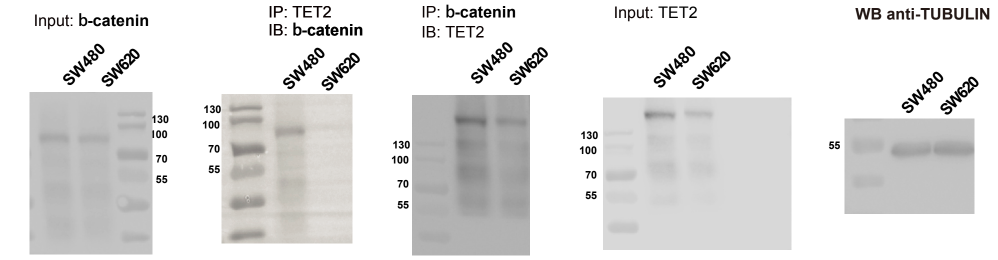

FigureS4 F

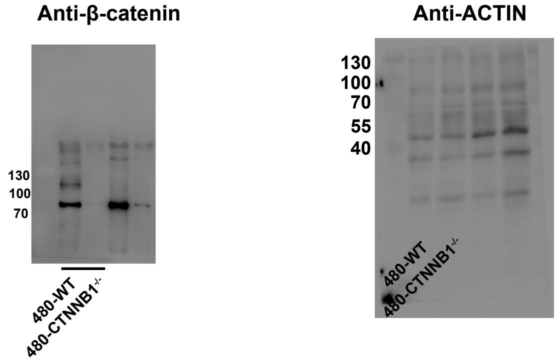

Figure S4K

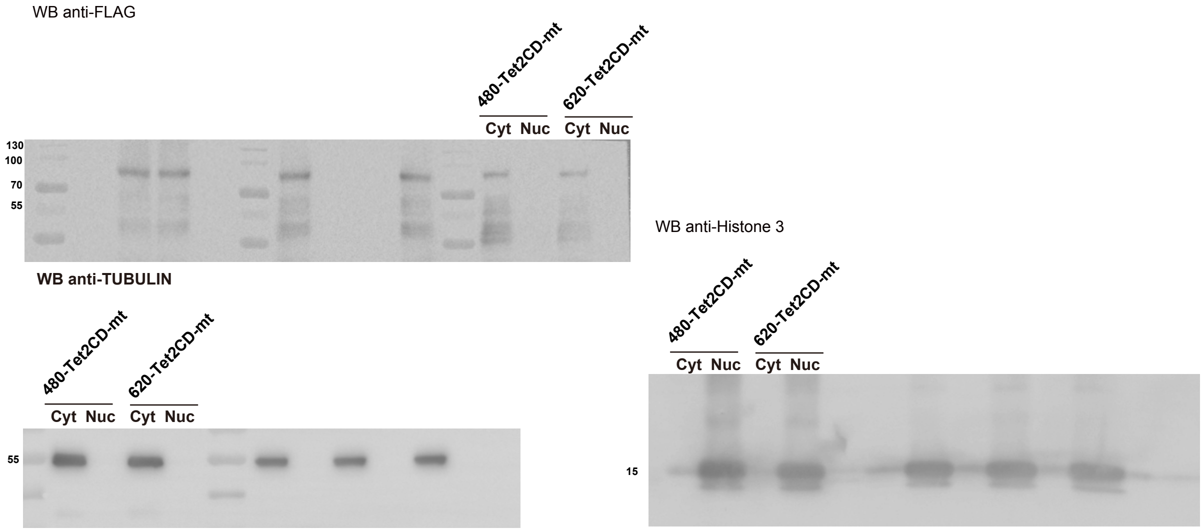

Figure S6C

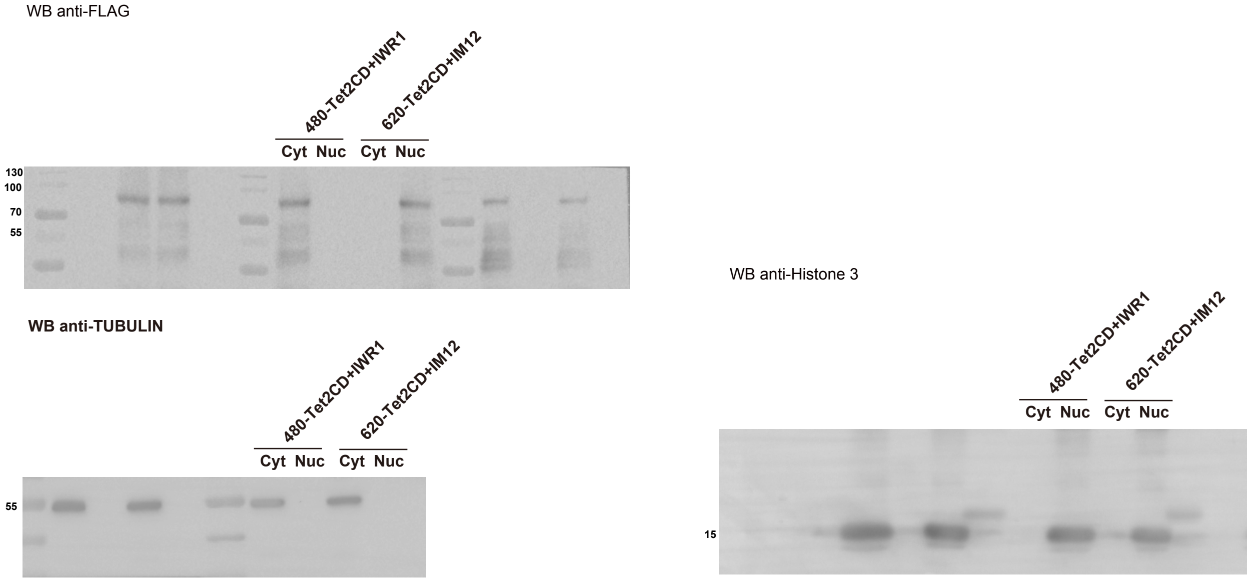

Supplement: Supplementary file 16 — original western blots [file 41419_2023_6038_MOESM16_ESM.pdf]
